# Enhancing the Water Solubility and Efficacy of Anticancer Drugs Using Hydroxypropyl-β-Cyclodextrin

**DOI:** 10.3390/ijms27020915

**Published:** 2026-01-16

**Authors:** Yasushi Kubota, Shinya Kimura

**Affiliations:** 1 Department of Clinical Laboratory Medicine, Saga-Ken Medical Centre Koseikan, Saga 840-8571, Japan; 2 Division of Hematology, Respiratory Medicine and Oncology, Department of Internal Medicine, Faculty of Medicine, Saga University, Saga 849-8501, Japan; shkimu@cc.saga-u.ac.jp

**Keywords:** cyclic oligosaccharide, cholesterol, cancer, leukemia, drug delivery system, active pharmaceutical ingredient, folate receptor, autophagy

## Abstract

Cyclodextrins (CyDs) are cyclic oligosaccharides that form inclusion complexes that allow organic compounds and other substances to be incorporated into their cavities. Hydroxypropyl-β-cyclodextrin (HP-β-CyD) is frequently used to improve the formulation properties of poorly water-soluble drugs because of its aqueous solubility and biocompatibility. Previous studies have demonstrated that the solubility and biocompatibility of poorly water-soluble anti-cancer agents can be improved by complexation with HP-β-CyD, which in some cases enhances their anticancer activity relative to the unmodified drugs. Advances in formulation strategies have enabled more efficient intracellular delivery, improved tissue and cell selectivity, and controlled release. HP-β-CyD has also been investigated as an active pharmaceutical ingredient, with demonstrated efficiency in treating leukemia and breast cancer. For example, folate-conjugated HP-β-CyD exhibits high selectivity for folate receptor-expressing cells and more potent anti-cancer activity than unmodified HP-β-CyD. Autophagy has been suggested to be involved in this mechanism. The continued development of drug-delivery systems that integrate advanced technologies and materials based on HP-β-CyD holds promise for further advances in cancer therapy. These findings indicate a paradigm shift in the role of HP-β-CyD from a formulation additive to an active pharmaceutical ingredient, suggesting broader applications for HP-β-CyD in anticancer treatments.

## 1. Introduction

The low aqueous solubility of many drug candidates presents a significant barrier to non-clinical developments and clinical applications. Numerous promising drug candidates, including those with anticancer activity, fail to reach patients due to poor water solubility. Therefore, improving solubility and developing appropriate formulations for hydrophobic drugs is a critical challenge in pharmaceutical development.

Cyclodextrins (CyDs) are cyclic oligosaccharides composed of glucose residues linked by α-1,4 bonds. Structurally, they possess a hydrophilic outer surface rich in OH groups and a hydrophobic region derived from CH groups on the inner surface. One characteristic of CyDs is their ability to form inclusion complexes, which allows them to incorporate organic compounds and other substances into their hollow structure [1]. CyDs are therefore widely used in the pharmaceutical industry to enhance drug solubility and bioavailability [2,3]. Among these, hydroxypropyl-substituted cyclodextrins (HP-CyDs) are being actively investigated for practical application in the fields of household goods and cosmetics, including sunscreen products [4]. In the pharmaceutical sector, HP-β-CyD is used as a pharmaceutical excipient to improve drug solubility in water and enhance safety [1,5].

CyDs also play an important role in the field of drug delivery [1,6,7,8,9]. Taking advantage of these characteristics, HP-β-CyD is being actively investigated for applications such as drug delivery systems (DDSs) for anticancer drug administration, as well as administration as active pharmaceutical ingredients (APIs) for intractable diseases and cancer. In addition, animal studies have been conducted to evaluate the toxicity (e.g., genotoxicity and carcinogenicity) of HP-β-CyD in a variety of animal species [10]. Investigations of pharmacokinetics and metabolic mechanisms have also been conducted, and the findings have shown that HP-β-CyD has low toxicity in humans [10]. This review summarizes the application of HP-β-CyD to encapsulate anticancer drugs to enhance solubility and antitumor activity. In addition, the status of HP-β-CyD as an API for cancer treatment is also assessed.

## 2. Cyclodextrin

**Figure 1:**
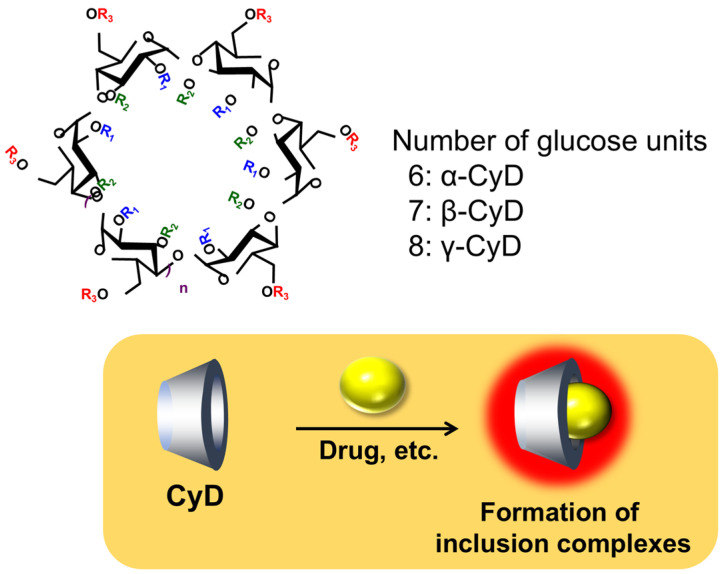
Structure and properties of cyclodextrin.

CyDs, obtained by applying enzymes to starch, were discovered by Antoine Villiers in the late 19th century and subsequently isolated and purified as cyclic oligosaccharides by Schardinger, Freudenberg, and others in the early 20th century [6]. CyDs, oligosaccharides formed by the cyclic linkage of glucose units via α-1,4 bonds, are classified into α-CyD (hexamer), β-CyD (heptamer), and γ-CyD (octamer) configurations based on the number of glucose units they contain (Figure 1) [11].

When CyDs are used to solubilize poorly soluble drugs, the drugs dissolve in the form of inclusion complexes formed with CyD, thereby improving their apparent solubility (Figure 1) [12,13]. Therefore, the solubility of drugs dissolved in CyDs depends on the solubility of CyD itself. The solubility of CyDs dissolved in water at 25 °C is 145 mg/dL for α-CyD, 18.5 mg/dL for β-CyD, and 232 mg/dL for γ-CyD. Particularly for β-CyD, its low water solubility has led to the synthesis and utilization of numerous derivatives with improved solubility (Table 1) [9,14,15].

Furthermore, CyDs are known to encapsulate low-molecular-weight substances within living organisms, with α-CyD encapsulating fatty acid chains and β-CyD encapsulating cholesterol [2]. Derivatives such as HP-β-CyD and sulfobutylether (SBE)-β-CyD have received FDA-approval as excipients for pharmaceutical applications; however, their use as single agents is not approved [16]. β-CyD holds promise for standalone pharmaceutical applications, with HP-β-CyD attracting particular attention for Niemann-Pick disease type C, a condition characterized by congenital accumulation of cholesterol within lysosomes [17,18,19,20,21,22,23]. In the field of cancer research, numerous in vitro and in vivo studies have demonstrated the encapsulation of poorly soluble anticancer drugs within HP-β-CyD to form inclusion complexes and thereby increase solubility, advancing efforts toward potential clinical application.

## 3. The Use of HP-β-CyD as a Solubilizing Excipient and Carrier System for Drug Delivery

Adequate stability and solubility in water are essential properties of drugs and drug candidates because they are closely related to bioavailability. β-CyD has been widely used as a pharmaceutical excipient because of its broad range of inclusion targets. However, the physical properties and functionalities of conventional CyDs have limitations, and developing new derivatives has been essential for their application in materials for DDSs, which require advanced functions. HP-β-CyD was developed in Europe and the United States as a solubilizer or stabilizer for parenteral formulations [5]. To increase the usefulness of compounds that exhibit anti-cancer activity, but whose clinical application is limited by their poor water solubility and low chemical stability, HP-β-CyD has been used as a complexation partner in inclusion complexes.

### 3.1. Cancer Prevention

HP-β-CyD has also been incorporated into sunscreens to prevent UV-induced skin cancer. Resveratrol-loaded polyvinylpyrrolidone/HP-β-CyD nanofibers showed more than 20,000-fold higher aqueous solubility than pure resveratrol and demonstrated excellent antioxidant activity and anti-inflammatory effects [24]. The ferulic acid (4-hydroxy-3-methoxy-cinnamic acid)/HP-β-CyD complex exhibited improved photostability and enhanced antioxidant activity compared with ferulic acid alone [25,26]. Hsu et al. showed that the ferulic acid/HP-β-CyD complex showed greater cytotoxicity toward hepatoma cell line Hep3B than ferulic acid alone, and conferred a stronger protective effect against CCl_4_-induced liver damage [27]. The antioxidant activity of three flavonols, i.e., kaempferol, quercetin and myricetin, increased upon complexation with HP-β-CyD [28]. Baicalein (BAI), a flavonoid isolated from *Scutellaria baicalensis*, possesses various pharmacological activities, including antioxidant, anti-inflammatory, and antitumor effects [29,30]. BAI was successfully complexed with HP-β-CyD using precipitation with a compressed antisolvent, resulting in improved solubility, antioxidant activity, and antibacterial activity [31]. Jullian et al. investigated complex formation of quercetin with three different CyDs (β-CyD, HP-β-CyD, and SBE-β-CyD) and found that the solubility of quercetin increased in the order β-CyD < HP-β-CyD < SBE-β-CyD, while the antioxidant activity was retained [32].

### 3.2. Cancer Treatment

This section introduces the current status of research aimed at enhancing water solubility and anticancer activity through the formation of complexes between HP-β-CyD and anticancer drugs. 

#### 3.2.1. Paclitaxel

Paclitaxel (PTX) is an anticancer drug that inhibits cell division by stabilizing microtubules, but it is highly hydrophobic. Therefore, to enable intravenous administration, solubilizing agents are required to increase its water solubility. This has led to interest in CyDs, which are expected to improve water solubility, reduce side effects, and enhance bioavailability [33,34]. To increase the oral bioavailability of PTX, formation of an inclusion complex with HP-β-CyD incorporated into poly(anhydride) nanoparticles (PTX-HP-β-CyD NPs) has been investigated [35,36]. Oral administration of PTX-HP-β-CyD-NPs maintained plasma PTX concentrations at a plateau close to Cmax for up to 24 h and exhibited cytotoxicity 33-fold higher than previously reported. Furthermore, the oral bioavailability of PTX was calculated to be approximately 80%. It is speculated that CyD-mediated inhibition of P-glycoprotein (P-gp) and cytochrome P450 enzymes contributes to this phenomenon [37,38]. Adding polyethylene glycol (PEG) to PTX-HP-β-CyD-NP increased intestinal permeability in rats in vitro by 10–15-fold compared to commercial Taxol^®^, and maintained plasma PTX concentrations in C57BL/6J mice for at least 24 h. The increased intestinal permeability and prolonged plasma PTX exposure produced by PEG-modified PTX–HP-β-CyD–NP resulted in a relative oral bioavailability of PTX of approximately 80% [39].

The potential of surface-modified PTX-incorporated solid lipid nanoparticles (SLNs) with HP-β-CyD (PSC) to improve PTX solubility and cellular uptake has also been investigated as an oral delivery system. The solubility of PSC was increased 16 to 17-fold compared to PTX (Table 2) [40]. The cytotoxicity and cellular uptake of PSC were significantly higher than those of PTX solution in Caco-2 and MCF-7 cells [40,41]. In particular, PSC was also effective against MCF-7/ADR cells, a multidrug-resistant breast cancer cell line expressing P-gp [41]. Treatment with PSC induced significantly more apoptotic cell death in MCF-7 cells than either PTX solution or PTX-incorporated SLNs, suggesting that HP-β-CyD enhances PTX-induced apoptosis [42]. The stability of PTX-loaded SLNs (PS) and PSC was evaluated by measuring encapsulation efficiency, particle size, polydispersity index, in vitro release profiles, and cytotoxicity. These parameters did not change between PS and PSC after 180 days of storage at 25 °C; however, when samples were stored at 40 ± 2 °C, PSC was more stable than PS, suggesting that HP-β-CyD confers additional stability to PS [43]. PSC also showed strong antitumor activity in vivo, and intravenous administration of PSC reduced nephrotoxicity compared with PTX solution containing Cremophor EL [42]. Furthermore, co-encapsulation of PTX with a first-generation P-gp inhibitor, verapamil [44], in SLNs using HP-β-CyD resulted in significantly higher cytotoxicity, cellular uptake, and downregulation of P-gp expression compared with the PTX solution in MCF-7/ADR cells [45].

HP-β-CyD–graphene oxide (GO) conjugates were designed and evaluated as PTX carriers. The PTX-containing GO-HP-β-CyD exhibited improved cytotoxicity against HeLa cells in vitro [46]. HP-β-CyD-(L-Arg)_2_-modified quantum dot (QD) NPs, designed to deliver Bcl-2 siRNA and PTX simultaneously, showed three- to four-fold greater cytotoxicity against A549 cells compared with PTX alone [47]. Attempts to overcome multidrug resistance (MDR) by incorporating the PTX/CyD complex into liposomes have also been reported using the PTX-resistant lung cancer cell line A549/T. Liposomes are widely used as DDSs because of their biodegradability, low toxicity, and ability to solubilize hydrophobic drugs [48,49]. PTX/HP-β-CyD complex-loaded liposomes (PTXCDL) significantly improved PTX solubility and anticancer activity in both in vitro and in vivo. Optimized PTXCD (prepared with a molar ratio of 1:10 and 0.5 mL of dehydrated alcohol) exhibited a solubility of 211.48 μg/mL, which is 556.5 times higher than that of free PTX (Tabel 2). Moreover, PTXCDL demonstrated superior cytotoxicity and intracellular uptake compared with PTXL, suggesting that HP-β-CyD may inhibit PTX efflux in A549/T cells [50]. 2-HP-β-CyD modification of PTX-loaded poly(lactide-co-glycolide) (PLGA) NPs enhanced PTX cytotoxicity and cellular uptake. Pharmacokinetic analysis demonstrated that the t_1/2β_ and area under the curve (AUC) values for 2-HP-β-CyD/PTX/PLGA NPs were significantly higher than those of plain PLGA NPs (t_1/2β_, 3.4-fold; AUC, 1.7-fold). Furthermore, these changes extended the systematic circulation time of 2-HP-β-CyD/PTX/PLGA NPs in vivo, with the particles remaining in the mouse lungs as long as 6 h after administration, suggesting potential utility for lung cancer treatment [51].

Another biodegradable polymer, poly-3-htdroxybutyrate (PHB), was also evaluated. HP-β-CyD-PTX/PHB NPs exhibited 2.6-fold greater cytotoxicity against MCF-7 cells than free PTX and strongly induced apoptosis and G2/M-phase arrest [52]. Nanocrystals (NCs) are attractive formulations for the delivery of anticancer drugs [53]. PTX release from HP-β-CyD-coated PTX NCs was significantly enhanced compared with free PTX or uncoated PTX NCs, a result attributed to both the presence of CyD and the physicochemical characteristics of the NCs [54,55]. Furthermore, blood hemolysis after intravenous injection was reduced with HP-β-CyD-coated PTX NCs. This is attributed to improved size and hydrophilicity resulting from HP-β-CyD coating, as well as faster PTX release from HP-β-CyD-coated PTX NCs compared to PTX NCs, thereby reducing exposure of crystalline PTX to blood components [56]. Methods for combining arginine and biotin with HP-β-CyD have also been investigated. PTX-loaded biotin-Arg(Pbf)-HP-β-CyD exhibited superior anticancer activity compared to PTX solution containing Cremophor EL, both in vitro and in vivo. Biotin-Arg(Pbf)-HP-β-CyD NPs are internalized into cells via biotin receptor-mediated endocytosis, thereby enhancing cellular uptake [57]. To further enhance the therapeutic efficacy of PTX-loaded biotin-Arg-HP-β-CyD, benzimidazole- and mannose-modified hyaluronic acid (BM-HA-Man) was employed as a pH-sensitive plug. The resulting PTX-loaded biotin-Arg-HP-β-CyD/BM-HA-Man inclusion complex effectively released PTX at the tumor site and reprogrammed M2 macrophages into antitumor M1 macrophages, an effect facilitated by HA. In vivo studies using 4T1 tumor-bearing mice showed marked tumor reduction following treatment with the PTX-loaded biotin-Arg-HP-β-CyD/BM-HA-Man inclusion complex [58].

#### 3.2.2. Camptothecin

Topoisomerase I is a key target in cancer chemotherapy, and camptothecin (CPT), an alkaloid derived from Camptotheca acuminata, was the first anticancer compound shown to inhibit topoisomerase I [59,60,61,62]. However, its clinical application has been hindered by low water solubility and poor chemical stability, prompting efforts to improve its stability and bioavailability. Several attempts to improve the solubility, stability, and anticancer activity of CPT through the formation of inclusion complexes with HP-β-CyD have been reported. The CPT/HP-β-CyD inclusion complex exhibited sub-micromolar cytotoxicity against several human cancer cell lines, namely AREc32 (breast cancer), H-23 (lung cancer), HepG2 (liver cancer), A2780 (ovarian cancer), and SH-SY5Y (neuroblastoma). The CPT/HP-β-CyD complex demonstrated approximately 2- to 7-fold higher potency than free CPT, depending on the cell line [63].

Nine-nitrocamptothecin (9-NC, Rubitecan) is an analog of CPT and is considered a highly promising anticancer agent demonstrating potent efficacy against a wide range of human cancers in preclinical setting [64]. Jiang et al. developed a 9-NC/HP-β-CyD complex using a co-lyophilization technique [65,66]. The solubility of the extremely hydrophobic compound 9-NC was dramatically improved by complexation with HP-β-CyD (from <5 μg/mL to 0.52 mg/mL) [65], and the resulting 9-NC/HP-β-CyD complex exhibited antitumor activity against ovarian, breast, and epithelial cervical cancer cells. In vivo administration of the 9-NC/HP-β-CyD complex demonstrated a significant tumor growth-inhibitory effect comparable to that of free 9-NC, while exhibiting lower toxicity, indicating an improved safety profile [66]. The antitumor activity of transferrin-9-NC/CyD-CL, in which the 9-NC/CyD complex was encapsulated into liposomes via ethanol injection and transferrin was conjugated to the liposomal surface, was further enhanced in both in vitro and in vivo experiments. In this study, the aqueous solubility of 9-NS/HP-β-CyD complex was increased 875-fold compared to free 9-NC (Table 2) [67].

#### 3.2.3. Doxorubicin

Doxorubicin (DXR) inhibits selective autophagy of peroxisomes (pexophagy) in neurons, increasing peroxisome numbers and enhancing the production of reactive oxygen species (ROS) [68]. Since HP-β-CyD activates transcription factor EB (TFEB), a transcription factor that regulates autophagy- and lysosomal function-related gene expression [68,69], its combination with DXR was shown to improve peroxisome clearance and related ROS production [70]. This finding suggests a potential therapeutic strategy for reducing the neurotoxicity and cognitive impairment caused by DXR. The complex of _D_-limonene and HP-β-CyD (HβDL) has also been reported to mitigate DXR-induced cardiotoxicity [71,72]. In mouse studies, HβDL prevented cardiomyocyte death by maintaining calcium homeostasis, reducing oxidative damage, and suppressing apoptosis pathways, while preserving the anticancer effects of DXR [72].

#### 3.2.4. Cisplatin

The efficacy of a local DDS using visible light-curable glycol chitosan (GC) hydrogel [73,74] and HP-β-CyD has been reported for the combined administration of cisplatin (CDDP) and DXR to treat osteosarcoma and reduce side effects. The resulting formulation (DXR-hydrochloride/CDDP-loaded visible light-cured glycol chitosan hydrogel/HP-β-CyD) demonstrated superior antitumor activity compared to either agent alone in in vitro and in in vivo studies, and systemic side effects were minimal with local administration [75]. Liu et al. reported that CyD-assisted extraction (using β-CyD or HP-β-CyD) is a technology that enhances the solubility and extraction efficiency of bioactive compounds such as flavonoids, polyphenols, and artemisinin from medicinal plants [76]. They developed an extraction method integrating HP-β-CyD assisted extraction and macroporous resin purification for the saponin total fraction derived from *Paris polyphylla var. yunnanensis* (PPT), whose individual saponin components have demonstrated anticancer effects against bladder cancer [77,78]. This method increased saponin purity by approximately 15-fold, and the purified PPT enhanced the chemotherapy sensitivity of CDDP-resistant bladder cancer cells [79].

#### 3.2.5. Venetoclax

BCL2 is an anti-apoptotic protein that regulates apoptosis by modulating mitochondrial membrane permeability [80]. It is highly expressed in tumor cells and contributes to treatment resistance [81]. Venetoclax (VEN) was developed as a BH3 mimetic that selectively inhibits BCL2 and has demonstrated potential in various cancers, including breast cancer and colorectal cancer, as well as acute myeloid leukemia [82,83,84]. However, the use of VEN has been limited by its rapid degradation, low aqueous solubility, and poor pharmacokinetic properties [85].

To overcome these limitations, a variety of nanocarriers have been investigated [86,87,88,89]. Chandani et al. prepared VEN-loaded HP-β-CyD NPs (VEN/HP-β-CyD NPs) and examined efficacy against triple-negative breast cancer (TNBC). VEN/HP-β-CyD NPs exhibited IC_50_ values 4–5 fold lower than free VEN in both 4T1 and MDA-MB-231 cells. This enhanced antitumor effect was attributed to improved intracellular uptake of VEN/HP-β-CyD NPs. Furthermore, in vivo efficacy in a 4T1-based TNBC model was significantly improved in the VEN/HP-β-CyD NP group compared to the free VEN group, with minimal systemic toxicity [90]. Another study reported that forming VEN/HP-β-CyD inclusion complexes by the kneading method enhanced aqueous solubility by up to 3.6-fold compared to pure VEN and improved antitumor activity against lung cancer cells (Table 2) [91].

#### 3.2.6. Curcumin

Curcumin is a natural polyphenolic compound that is the main component of turmeric and its primary coloring agent [92,93]. Curcumin has been studied for decades as a potential anticancer agent, with mechanisms of action that include apoptosis induction, inhibition of cell proliferation, and modulation of signaling pathways [94,95]. Curcumin has demonstrated anticancer activity against human colorectal cancer [96,97,98], but its low water solubility and bioavailability have hindered the development of colon-targeted DDSs. To overcome these limitations, Jyoti et al. enhanced the solubility of curcumin to the colon by complexing curcumin with HP-β-CyD and then fusing the complex into chitosan microspheres to enable selective delivery of curcumin to the colon [99]. In another study, the curcumin-HP-β-CyD inclusion complex exhibited a higher IC_50_ value against the MCF-7 breast cancer cell line than curcumin alone, indicating reduced cytotoxic potency [100].

#### 3.2.7. Other Agents

Resveratrol (RV, 3,4′,5-trihydroxy-trans-stilbene) is a phytoalexin produced by plants in response to parasitic attack or environmental stress, and its antitumor effects against many tumor cell lines have been widely studied [101,102]. The chemopreventive activity of topical RV-HP-β-CyD complexes (cream and mouthwash formulations) was evaluated using both in vitro and in vivo oral squamous cell carcinoma (OSCC) models. RV-HP-β-CyD complexes significantly inhibited OSCC development compared with the RV-ethanol treatment group [103]. The efficacy of the RV/HP-β-CyD complex has also been demonstrated in a cervical cancer model. The aqueous solubility of the RV/HP-β-CyD complex was approximately 440-fold higher than that of free RV, and the RV/HP-β-CyD complex significantly reduced and prevented tumor growth compared with free RV [104]. It has also been reported that naturally occurring resveratrol analogs with potent anti-cancer activity, including Z-3,5,4′-trimethoxystilbene, trans-2,6-difluoro-4′-N,N-dimethylaminostilbene, trans-4,4′-dihydroxystilbene, and gnetol, exhibit improved water solubility and stability upon inclusion in HP-β-CyD [105,106,107,108]. More recently, a composite nanogel system that co-encapsulates DXR and RV has been reported to reduce DXR-induced cardiotoxicity and neurotoxicity in vitro. In this system, a RV-HP-β-CyD complex was first prepared at a 1:10 molar ratio, after which double-loaded nanogel particles were formulated. The encapsulation efficiency of RVin the nanogel reached 97.8%, exceeding that of several previously reported NP systems [109].

Photodynamic therapy (PDT) is a selective treatment method in which a tumor-tropic photosensitizing agent is administered and the tumor tissue is subsequently irradiated with a laser to induce a photochemical reaction, thereby causing necrosis of the tumor tissue [110]. Fullerene C_60_ is widely recognized as an efficient photosensitizer for PDT [111]. C_60_/HP-β-CyD NPs induced cytotoxicity by generating ROS upon exposure to visible light in vitro [112,113] and significantly inhibited tumor growth in mice bearing subcutaneously transplanted S-180 sarcoma cells [114].

Saikosaponin is a triterpenoid saponin isolated from plants in genus Bupleurum and has been traditionally used in medicine in China and Japan [115]. In particular, saikosaponin-D (SSD) has been reported to exert antioxidant, anti-inflammatory, and anticancer effects against various types of cancer [116,117]. SSD-HP-β-CyD complexes were synthesized at molar ratios of 1:1, 1:5, and 1:10, with the 1:5 complex exhibiting approximately 1074-fold greater aqueous than pure SSD (827.52 µg/mL). These complexes induced cytotoxicity in HSC-1 human cutaneous squamous cell carcinoma cells via apoptosis (Table 2) [118].

Inclusion complexes of barbigerone (2′,4′,5′-trimethoxy-6′′,6′′-dimethylpyrano(2′′,-3′′:7,8)-substituted flavonoid), an indole-substituted dipyrido [2,3-d]pyrimidine derivative, or a spirooxindole-pyrrolizidine derivative with HP-β-CyD have been generated for the purpose of developing therapeutic agents for hepatocellular carcinoma (HCC) in vitro and in vivo [119,120,121].

Albendazole (ABZ), a microtubule depolymerizing agent, displays marked anticancer activity against various cancer cell types [122,123,124]. The combination of ABZ, acetic acid, and HP-β-CyD significantly enhanced its aqueous solubility (10,368-fold compared to free ABZ), pharmacokinetic properties, and anti-tumor efficacy (Table 2) [125]. N,N′-bis-naphthyl 2-alkyl-substituted imidazolium salts exhibit high anticancer activity against several non-small cell lung cancer (NSCLC) cell lines, but their limited water solubility makes systemic administration difficult. Complexation with HP-β-CyD enabled dissolution at concentrations of up to 4.4 mg/mL [126].

Chrysin, a bioflavonoid, possesses multiple biological activities, including antioxidant, anti-inflammatory, and anticancer effects; however, its bioavailability is limited by poor aqueous solubility. Complexes of chrysin with several CyDs, including β-CyD, HP-β-CyD, SBE-β-CyD, and randomly methylated-β-CyD (RAMEB), were prepared, and their solubilities and cytotoxicities were evaluated in Caco-2 adenocarcinoma cells. Chrysin exhibited the greatest cytotoxicity when complexed with RAMEB, but none of the chrysin-CyD complexes showed cytotoxicity in Caco-2 cells at concentrations up to 100 μM (Table 2). At 200 μM, cell viability decreased significantly [127]. Another flavonoid, fisetin (FST; 2-(3,4-dihydroxyphenyl)-3,7-dihydroxychromen-4-one), also displays cytotoxic activity against various cancers [128,129,130,131,132,133,134,135]. The solubility of FST increased from 5.25 μg/mL to 850 μg/mL after forming an inclusion complex with HP-β-CyD (Table 2). An FST-HP-β-CyD inclusion complex (FHIC) was further encapsulated in poly-lactide-co-glycolic acid NPs (PLGA NPs), and the bioavailability of orally administered FHIC-PLGA NPs was compared with that of pure FST in C57BL/6 mice. FHIC-PLGA NPs significantly enhanced the anticancer activity of FST, inducing apoptosis in MCF-7 human breast cancer cells [136].

More recently, the therapeutic efficacy of a complex of purinostat mesylate (PM), a novel histone deacetylase inhibitor, and HP-β-CyD (hereafter PM/HP-β-CyD) was reported [137]. Although PM has demonstrated efficacy against B-cell-associated lymphomas, its poor solubility has hindered clinical application [138]. To address this limitation, PM/HP-β-CyD was generated, increasing the solubility of PM by approximately 220-fold. PM/HP-β-CyD maintained in vitro antitumor activity comparable to that of free PM and showed greater efficacy and lower toxicity than rituximab plus hyper-CVAD or LBH589 in vivo [137].

Resiquimod (R848), a Toll-like receptor 7 agonist, can polarize tumor-associated macrophages from an M2-like phenotype to an M1-like phenotype [139,140]. However, R848 rapidly diffuses systemically and causes toxicity due to its pharmacokinetic behavior [141,142]. To address this challenge, R848 was encapsulated in an FDA-approved biodegradable polymer, poly(D,L-lactic-co-glycolic acid) (PLGA) NPs and subsequently modified with HP-β-CyD [143]. Furthermore, HP-β-CyD has been reported to exhibit affinity for macrophages [51]. As a result, R848-loaded PLGA NPs modified with 2-HP-β-CD (CD@R848@NPs) showed enhanced penetration into tumor tissue, dramatically reprogrammed macrophages toward M1-like macrophages, eliminated the tumor, and significantly prolonged survival in tumor-bearing mice [143].

Amygdalin (AMY) is a natural anticancer compound derived from the fruit kernels of plants in the Rosaceae family [144], and it has been shown to inhibit the growth of prostate, leukemia, breast, colon, and bladder cancer cells [145,146,147,148,149]. An AMY/HP-CyD inclusion complex reached its maximum release (approximately 100%) within 4 h and significantly inhibited the proliferation of HeLa cancer cells (CCL-2) compared with AMY alone [150].

Dasatinib is a second-generation ABL tyrosine kinase inhibitor that exhibits approximately 325-fold stronger inhibition of BCR-ABL in vitro compared to imatinib [151,152]. Recently, dasatinib has also been suggested as a potential therapeutic agent for Duchenne muscular dystrophy, owing to its inhibition of SRC kinase. Encapsulation of dasatinib with HP-β-CyD enables preparation of an aqueous formulation of this otherwise water-insoluble drug, facilitating both oral and parenteral administration. The dasatinib/HP-β-CyD inclusion complex has therefore been proposed as a promising therapeutic tool for pediatric Duchenne muscular dystrophy patients [153]. Another research group synthesized inclusion complexes of dasatinib and CyDs using a mechanochemical method with the aim of improving solubility, controlled release, and overall bioavailability. Molecular docking simulations revealed that β-CyD is the most suitable host for dasatinib among the three natural CyDs (α, β, and γ-CyD), and in vitro release studies using tablets containing HP-β-CyD complexes showed improved dasatinib release profiles compared with untreated dasatinib [154]. However, to determine whether the dasatinib/HP-β-CyD complex is effective in treating leukemia, its pharmacokinetic behavior and therapeutic activity must be confirmed in vivo.

Sotorasib (AMG-510) [155,156], a drug approved for the treatment of NSCLC harboring KRAS G12C mutations, requires high-dose administration because its oral bioavailability is only approximately 20%. Patel et al. developed a novel freeze-dried nanocrystal formulation of sotorasib using a Quality by Design approach to improve solubility and bioavailability. Trehalose and HP-β-CyD were identified as the most effective protective agents for maintaining NC stability during freeze-drying, with HP-β-CyD preserving a consistent particle-size distribution under freezing conditions [157].

Thymoquinone (TQ), a compound extracted from *Nigella sativa*, has been reported to exhibit anticancer activity against several tumor types, including NSCLC. However, its low aqueous solubility limits clinical application [158,159,160]. Zheng et al. demonstrated that encapsulation of TQ within HP-β-CyD increased its aqueous solubility by 1559-fold and conferred substantially greater anticancer activity against A549 and HCC827 cells than free TQ (Table 2). The proposed mechanism involved induction of ferroptosis via suppression of NF-κB activity. Furthermore, in vivo experiments using the A549 xenograft mouse model demonstrated potent antitumor effects and evidence of ferroptosis, consistent with the in vitro findings [161].

**Table 2:** List of in vitro studies on anticancer drugs that demonstrated both improved water solubility and enhanced anticancer activity by forming complexes with HP-β-CyD.

Drug	Additional Agent	Synthesis	Solubility *	Type of Cancer	In Vitro	Anticancer Effects	Reference
Paclitaxel		Hot sonication, lyophilization	16–17.2	Colon	Caco-2	Increased cytotoxicity	[40]
Paclitaxel		Hot sonication, lyophilization	16–17.2	Breast	MCF-7, MCF-7/ADR	Increased cytotoxicity and cellular uptake	[41]
Paclitaxel		Hot sonication, lyophilization	16–17.2	Breast	MCF-7	Improved anticancer activity and cellular uptake	[42]
Paclitaxel		Aqueous solution-stirring method	47.1–556.5	Lung	A549/T	Enhanced cytotoxicity and cellular uptake	[50]
9-nitro-camptothecin	Transferrin, liposome	Lyophilization	875	Ovarian, liver	HepG2, A2780, L02	Enhanced cytotoxicity	[67]
Venetoclax		Kneading	3.16	Lung	A549	Enhanced cytotoxicity	[91]
Saikosaponin-D		Stirring, lyophilization	351–1074	Cutaneous	HSC-1	Apoptosis	[118]
Albendazole		Stirring	10,368	Colorectal, prostate	HCT116, DU145	Antiproliferative effect	[125]
N,N′-bis-naphthyl 2-alkyl-substituted imidazolium salts		N.D.	>8.8	Lung	NCI-H460, NCI-H1975, NCI-A549, HCC827	High anticancer activity comparable to cisplatin	[126]
Chrisyn		Lyophilization	5.66–7.52 (RAMEB > SBECD > HP-β-CyD > β-CyD) **	Colon	Caco-2	No cytotoxicity up to 100 µM	[127]
Fisetin	PLGA	Coacervation technique, physical mixture	161.9	Breast	MCF-7	Higher cytotoxicity, increased cellular uptake	[136]
Purinostat		Simple dissolution	220	B-cell lymphoma	SU-DHL-6	Maintained cytotoxicity	[137]
Thymoquinone		Lyophilization	1559	Lung	A549, HCC827	Enhanced cytotoxicity	[161]

* These values represent the solubility of the complex with HP-β-CyD when the solubility of the free drug is set to 1. ** Among the β-CyDs examined, solubility was highest in the order RAMEB, SBECD, HP-β-CyD, β-CyD. PLGA, poly(lactide-co-glycolide).

Targeting both autophagy inhibition and mitochondrial fission holds promise for the treatment of TNBC, which currently lacks effective options [162,163]. One such agent is 5-(4-hydroxyphenyl)-3H-1,2-dithiole-3-thione (ADT-OH), a sustained-release hydrogen sulfide donor; however, its low membrane permeability and poor aqueous solubility necessitate high doses to achieve therapeutic concentrations [164,165]. The inclusion complex of ADT-OH with HP-β-CyD (CD-ADT-OH) exhibited improved aqueous solubility and demonstrated superior anticancer effects compared with free ADT-OH. CD-ADT-OH also produced significant antitumor effects in vivo at a low dose (10 mg/kg). Furthermore, in both the MDA-MB-231 orthotopic xenograft model and the 4T1-Luci tail-vein metastasis model, CD-ADT-OH suppressed metastasis without significantly affecting mouse body weight, confirming its safety [166].

In vitro studies on HP-β-CyD–drug complexes with anticancer activity are summarized in Table 2, Table 3 and Table 4. Table 2 lists drugs whose water solubility and anticancer activity increased upon forming inclusion complexes with HP-β-CyD. Meanwhile, Table 3 and Table 4, respectively, list drugs for which improved water solubility was observed but anticancer activity was not investigated, and drugs for which increased anticancer activity was observed but changes in water solubility were not described. Table 5 summarizes in vivo studies investigating the anticancer activity of HP-β-CyD–drug complexes.

## 4. HP-β-CyD as an Active Pharmaceutical Ingredient

In addition to serving as a drug solubilizer, stabilizer, and DDS carrier, HP-β-CyD itself has been reported to exert therapeutic activity. Water-soluble CyD derivatives can be used to prepare highly concentrated aqueous solutions and are generally safe. In addition, various substituents can be introduced into the hydroxyl groups of the glucose units that constitute the CyD molecule, allowing for rational and modular molecular design. Recently, attempts to exploit the biological effects induced by interactions between CyDs and biomembrane components for disease treatment have attracted considerable attention, and a paradigm shift is emerging in which CyDs are being developed as APIs rather than excipients. For example, sugammadex, in which all primary hydroxyl groups of γ-CyD are carboxythioether-modified, forms an extremely strong inclusion complex with the steroidal neuromuscular blockers rocuronium and vecuronium, exhibiting excellent antagonistic activity [167,168,169]. This represented the first example of a CyD derivative approved for use as an API [170], renewing momentum to develop additional CyD-based APIs.

Niemann-Pick disease type C (NPC) is a deficiency of NPC1 and NPC2, proteins that transport cholesterol and glycolipids from endosomes and lysosomes to the cytoplasm and cell membrane. Loss of NPC1/NPC2 function leads to intracellular accumulation of cholesterol and sphingomyelin, resulting in hepatosplenomegaly and progressive central nervous system damage [171]. In an exploratory study on the treatment of NPC, HP-β-CyD, which was initially used as a solubilizer for a candidate compound (allopregnanolone), was found to improve disease symptoms in *Npc1*-deficient mice [17,172]. Based on these results, compassionate-use administration of HP-β-CyD was approved for patients with NPC [173,174,175]. In 2013, a phase 1/2a clinical trial of intrathecal HP-β-CyD began in the United States, followed by a phase 2b/3 trial in 2016 [20,176].

Cholesterol accumulation and/or dysregulated cholesterol homeostasis have also been reported in various malignancies, including leukemia and breast cancer [177,178,179,180,181,182,183,184,185,186]. Therefore, the modulation of cholesterol homeostasis represents a reasonable strategy for anticancer drug development [187,188]. Experiments using BCR-ABL-positive cell lines showed that HP-β-CyD treatment reduced intracellular cholesterol levels and significantly inhibited leukemic cell growth through G_2_/M cell cycle arrest and induction of apoptosis. Furthermore, intraperitoneal administration of HP-β-CyD significantly improved survival in a BCR-ABL leukemia mouse model [189].

Subsequent to this report, several studies have investigated the anticancer activity of HP-β-CyD as a standalone agent in breast cancer models [190,191,192,193]. In MDA-MB-231 breast cancer cells, HP-β-CyD was reported to suppress the epithelial–mesenchymal transition by inhibiting transforming growth factor-β/Smad signaling and introducing endoplasmic reticulum stress [190,191]. Saha et al. examined the effect of cholesterol depletion in the treatment of TNBC using HP-β-CyD. Treatment with HP-β-CyD induced cholesterol depletion in TNBC cells, resulting in growth inhibition through apoptosis in vitro, and also produced tumor-reducing effects in vivo in TNBC xenograft mouse models [192]. Zhu et al. also demonstrated that HP-β-CyD exhibits anti-proliferative and anti-migratory effects in TNBC cells in vitro and in vivo. The proposed mechanism involves HP-β-CyD simulating cholesterol efflux through ABCA1 and ABCG1, thereby reducing cholesterol levels within tumor cells [193]. Taken together, these results suggest that HP-β-CyD has potential as an anticancer drug when used as an API.

## 5. Folate-Appended Cyclodextrins

To enhance the accumulation of anticancer drugs at tumor sites and improve therapeutic efficacy, ligands can be conjugated to nanocarriers. This strategy facilitates drug uptake into tumor cells by exploiting specific interactions between the ligand and its corresponding receptor [194,195]. In recent years, DDSs based on folic acid (FA) and folate receptor α (FRα) have been developed for cancer diagnosis and therapy. FRα is expressed at very low levels in most normal cells, but is highly overexpressed in many cancer cell types, except for A549 cells [196,197]. In addition to FRα, the reduced folate transporter (RFC; also known as SLClgAl) is a major transporter responsible for folic acid uptake into both normal and cancer cells [198]. However, RFC cannot transport folate-conjugated drugs into cells [197]. As a result, folate-appended agents exhibit minimal effects on normal cells while enabling selective uptake by FRα-overexpressing cancer cells. Thus, when a folate-conjugated anticancer agent is administered to a patient with cancer, the drug can be delivered more efficiently to tumor tissue, and numerous folate-appended therapeutics are currently under development.

**Figure 2:**
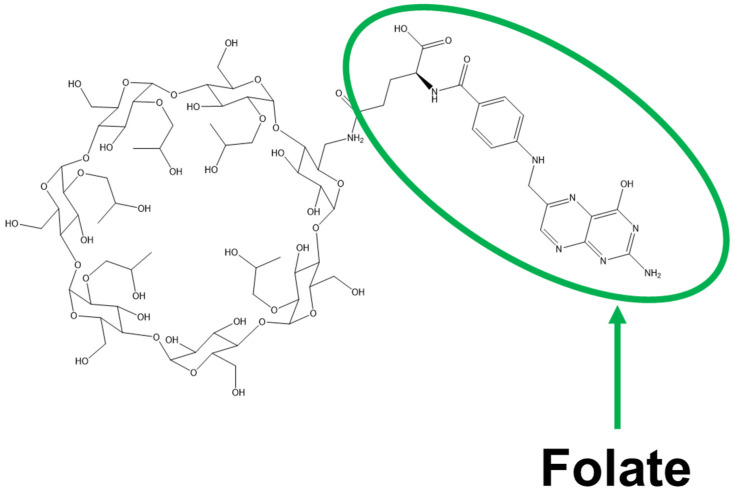
Folate-appended hydroxypropyl-β-cyclodextrin (FA-HP-β-CyD).

**Figure 3:**
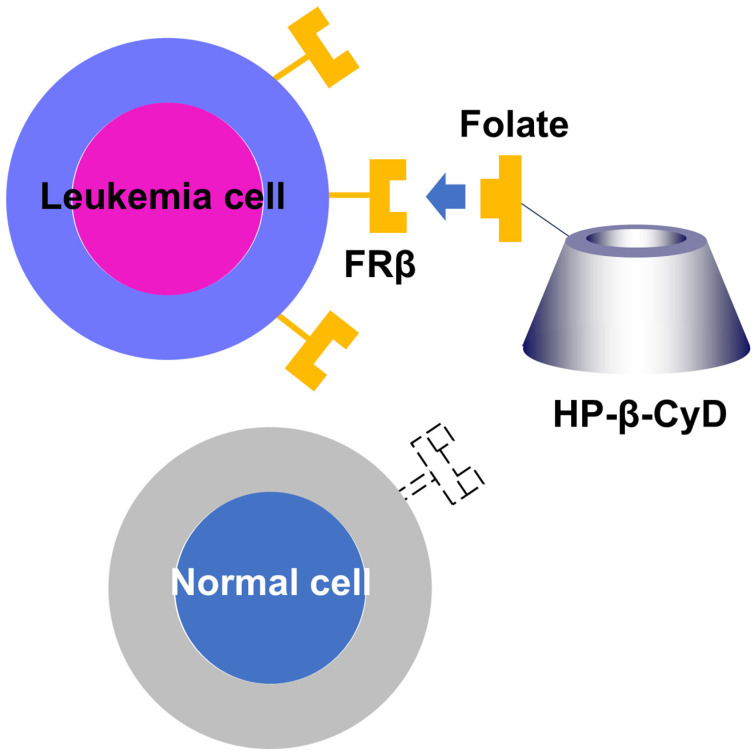
FA-HP-β-CyD interacts with leukemia cells via the folate receptor β (FRβ), but not with cells with low FR expression. FA-HP-β-CyD inhibits cell growth by apoptosis and autophagy-dependent cell death.

We previously showed that HP-β-CyD disrupts cholesterol homeostasis and inhibits the proliferation of leukemia cells by inducing apoptosis and cell-cycle arrest [189]. To confer tumor cell-selectivity on HP-β-CyD, we synthesized FA-HP-β-CyD (Figure 2) and evaluated the potential of FA-HP-β-CyD as an anticancer agent using BCR-ABL-positive leukemia cells (Figure 3).

The mechanism underlying the cell growth-inhibitory effect of FA-HP-β-CyD may involve autophagy, similar to FA-M-β-CyD [199,200,201,202]. The combination of FA-HP-β-CyD with tyrosine kinase inhibitors (imatinib and ponatinib) produced a synergistic inhibitory effect in BCR-ABL-positive leukemia cells. In a mouse model of BCR-ABL-induced leukemia, FA-HP-β-CyD exhibited a stronger inhibitory effect on leukemia progression than either HP-β-CyD or imatinib alone [203]. FA-HP-β-CyD also exhibited antileukemic activity in acute myeloid leukemia cells via autophagic cell death and showed synergistic activity with venetoclax, a BCL-2 inhibitor [204]. As therapeutic strategies targeting autophagy are being actively explored in hematologic malignancies, including leukemia, further investigation of the relationship between FA-HP-β-CyD and autophagy is warranted [205].

Although both FA-HP-β-CyD and FA-M-β-CyD exhibited no growth-inhibitory activity in FR-negative cells [199,203], paclitaxel formulated in a folate-appended β-CyD (PTX/Fol-c_1_-β-CyD) induced cytotoxicity in both FRα-positive epithelial ovarian cancer cells and FRα-negative cells. In vitro and in vivo studies showed that the activity of PTX/Fol-c_1_-β-CyD against FRα-negative A2780 cells was mediated by the proton-coupled folate transporter [206]. Further studies are needed to clarify how the selectivity of folate-modified CyDs differs among CyD derivatives. Future studies should elucidate the molecular mechanisms underlying FA-HP-β-CyD-induced cell death and assess the safety and efficacy of FA-HP-β-CyD for clinical application.

Research has also been conducted on enhancing the efficacy of DXR using FA-HP-β-CyD to overcome multidrug resistance [207]. By conjugating HP-β-CyD and FA to polyethylenimine (PEI), DXR can be encapsulated within the HP-β-CyD cavity, while Bcl-2 siRNA binds to the complex through the positive charge of PEI. The resulting FA-HP-β-CyD-PEI/DOX/siRNA formulation demonstrated higher intracellular uptake and stronger antitumor activity against DXR-resistant MCF-7/Adr cells compared with free DXR or non-FA-modified constructs, suggesting that FA modification enhances tumor-targeting capability [207]. Studies on FA-HP-β-CyD are summarized in Table 6.

## 6. Pharmacokinetic Profile

Regarding the clearance of HP-β-CyD, when administered intravenously to rats and dogs, the plasma half-life is very short at 0.4 h in rats and 0.8 h in dogs [10]. To establish optimal dosing conditions for HP-β-CyD, pharmacokinetic parameters were calculated for intravenous infusion in a patient with NPC. HP-β-CyD (2650 mg/kg) was administered by continuous intravenous infusion over 8 h, and serum deposition was modelled using a one-compartment pharmacokinetic model [208]. The resulting systemic concentrations were comparable to those achieved in vivo HP-β-CyD treatment in BCR-ABL leukemia mouse models [189]. Serum HP-β-CyD concentrations at steady state remained within the optimal range (0.1–2 mM) for HP-β-CyD efficacy in both in vitro and in vivo NPC models.

**Figure 4:**
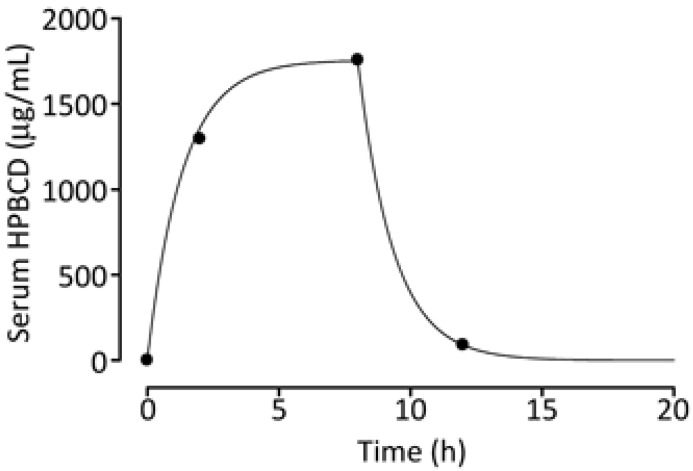
Changes in serum HP-β-CyD concentration in an NPC patient. The observed values for serum HB-β-CyD concentrations are shown by the points on the line. The simulated concentration–time curves serum HB-β-CyD concentration were estimated using the least-squares method and are shown by the solid line. The estimated pharmacokinetic parameters for the patient were as follows: systemic clearance, 198 mL/h/kg; volume of distribution, 266 mL/kg; and elimination half-life, 0.92 h. Reproduced from [208] with permission from the Pharmaceutical Society of Japan.

The volume of distribution (266 mL/kg) and clearance (198 mL/h/kg) of HP-β-CyD approximated the estimated extracellular fluid volume (250 mL/kg) and estimated glomerular filtration rate (175 mL/h/kg) for healthy children (Figure 4) [208].

These results suggest that, even when HP-β-CyD is administered at high doses to patients with NPC, as reported previously [209], HP-β-CyD is rapidly distributed within the extracellular fluid after intravenous administration and is cleared from the circulation at a rate comparable to glomerular filtration.

## 7. Toxicity

HP-β-CyD has been determined to be safe for parenteral administration and has been incorporated into commercial products [210]. Studies using model compounds have shown that parenteral administration of HP-β-CyD results in only a slight increase in drug-related toxicity [211]. Although the toxicity of HP-β-CyD is generally considered to be limited, its safely profile may vary depending on the route of administration [7,10,15]. Pitha et al. reported that HP-β-CyD itself is non-toxic and does not enhance the toxicity of tested compounds, but noted that increased solubility of co-administered drugs may influence their toxic effects due to rapid systemic distribution [5,212]. In animal studies, mice treated with HP-β-CyD showed no gross lesions on macroscopic examination, and neither hemolysis nor anemia was observed. More recently, lung toxicity associated with HP-β-CyD has received attention in the treatment of patients with NPC disease [173,213]. However, HP-β-CyD-treated mice showed no obvious histological abnormalities in the lungs [189], and HP-β-CyD administration had little or no measurable effect on pulmonary function [214,215]. In contrast, all mice injected intraperitonially with 150 mM M-β-CyD died of diffuse alveolar hemorrhage within 24 h [189]. HP-β-CyD appears to be the most promising candidate for the treatment of NPC; however, ototoxicity has emerged as a clinically relevant concern, particularly the development of hearing loss during treatment [216,217]. It has been reported that administration of HP-β-CyD causes abnormalities in auditory brainstem response testing and leads to the loss of inner ear hair cells in small animals, suggesting that HP-β-CyD induces auditory dysfunction by causing organic damage to inner ear hair cells [217,218]. Although the mechanism of HP-β-CyD-induced damage to inner ear hair cells is not clear at this time, it has been suggested that HP-β-CyD may affect the interaction between prestin, a motor protein expressed in hair cells, and intracellular cholesterol [219].

After one week of administration of 1-, 3-, and 5-fold the concentrations shown to be effective via systemic administration, HP-β-CyD caused decreases in the white blood cell count and body weight at the 5-fold dose, whereas FA-HP-β-CyD produced no detectable toxicity even at the highest concentration tested [204].

## 8. Barriers to Clinical Application

Therapeutic approaches using HP-β-CyD could become a technology enabling improved physicochemical stability, extended shelf life of anticancer drugs, support for targeting only tumor sites without damaging normal tissue, and maintenance of anticancer drug time profiles. However, several concerns exist that hinder the clinical application of HP-β-CyD as a drug delivery platform aimed at enhancing water solubility and anticancer activity, HP-β-CyD as an API, and folate-modified HP-β-CyD.

HP-β-CyD administered intravenously is excreted by the kidneys [10,220]. At doses exceeding a certain threshold, swelling and microvesiculation are observed in the renal cortical tubules and urinary tract uroepithelial cells [221]. This resembles osmotic nephropathy seen after intravenous administration of hypertonic glucose solutions, dextran, or plasma expanders [222,223]. Elimination of HP-β-CD is highly dependent on renal clearance; therefore, patients with renal dysfunction have a potential risk of HP-β-CD accumulation.

Because of rapid renal clearance and small volume of distribution values, the half-life (t½) of HP-β-CD is very short [10,220]. This is problematic when considering the efficacy of anticancer drugs; that is, HP-β-CD-containing anticancer drugs may fail to reach blood concentrations sufficient to exert adequate antitumor effects, potentially making it difficult to achieve the expected efficacy. To overcome this, a system that controls drug release is crucial, but drug delivery using HP-β-CyD alone makes it difficult to control drug release. Therefore, the development of formulations incorporating other carriers that can promote controlled drug release and accumulation within target tissues is essential [224].

HP-β-CyD has been reported to cause serious adverse events such as lung damage and ototoxicity in previous basic and clinical research and clinical trials [20,213,219,225]. Irie and colleagues found that HP-γ-CyD demonstrated equivalent NPC disease improvement effects to HP-β-CyD through drug screening using induced pluripotent cell lines established from NPC patients [226]. Moreover, it has been demonstrated that HP-γ-CyD exhibits lower toxicity to the lungs and hearing than HP-β-CyD [227]. However, it remains unknown whether HP-γ-CyD will exhibit antitumor activity comparable to or superior to that of HP-β-CyD when forming complexes with anticancer drugs.

It should be noted that combining HP-β-CyD with anticancer drugs does not necessarily enhance anticancer activity. Some reports indicate that anticancer activity remains unchanged compared to free drugs, or is even reduced [100,119,137]. The mechanism by which HP-β-CyD reduces antitumor activity by forming complexes may be explained by evidence suggesting either or both of the following: a potential reduction in the cytotoxic site within the anticancer agent, or HP-β-CyD protecting cell lines from the abrupt local high concentration of free anticancer drug [228,229]. More detailed verification and refinement of these challenges, along with further integration with other delivery systems, are desired to facilitate the transition from preclinical research to human clinical studies.

## 9. Conclusions

Numerous efforts have been made to combine anticancer drugs with HP-β-CyD, yielding promising in vitro results. However, in vivo studies and safety evaluations remain limited, and actual clinical translation has not yet been realized [230]. HP-β-CyD contributes to improvements in therapeutic development across various fields, including targeted anticancer agents, nucleic acid therapeutics, generic drug formulation, and applications in over-the-counter drugs, quasi-drugs, and health foods. The inventive use of HP-β-CyD to enhance the performance and value of pharmaceutical agents is expected to make a substantial contribution to future advances in medicine.

## Figures and Tables

**Table 1:** Characteristics of natural cyclodextrins and their derivatives.

Type of CyD	Substituent (R)	Mol. Wt. (Da)	Solubility (mg/mL)	Cavity diameter (Å)
Natural CyDs				
α-CyD		972	145	4.5–5.3
β-CyD		1135	18.5	6.0–6.5
γ-CyD		1297	232	7.5–8.3
Chemically modified CyDs				
HP-α-CyD	˗CH_2_˗CHOH˗CH_3_	1180	–	4.5–5.3
HP-β-CyD	˗CH_2_˗CHOH˗CH_3_	1400	>1200	5.8–6.5
CM-β-CyD	˗CH_2_˗CO_2_H	1541	50	–
DM-β-CyD	˗CH_3_	1331	570	5.8–6.5
RM-β-CyD	˗CH_3_	1312	>500	–
TM-β-CyD	˗CH_3_	1430	310	4–7
HE-β-CyD	˗CH_2_-CH_2_OH	1443	>2000	–
SBE-β-CyD	(CH_2_)_4_-SO_3_Na	2163	>1200	5.8–6.5
HP-γ-CyD	˗CH_2_˗CHOH˗CH_3_	1576	800	7.5–8.3

CyD, cyclodextrin; HP-α-CyD, hydroxypropyl-α-CyD; HP-β-CyD, hydroxypropyl-β-CyD; CM-β-CyD, carboxymethyl-β-CyD; DM-β-CyD, dimethyl-β-CyD; RM-β-CyD, randomly methylated-β-CyD; TM-β-CyD, trimethyl-β-CyD; HE-β-CyD, hydroxyethyl-β-CyD; SBE-β-CyD, sulphobutylether-β-CyD; HP-γ-CyD, hydroxypropyl-γ-CyD.

**Table 3:** List of in vitro studies on anticancer drugs that demonstrated improved water solubility by forming complexes with HP-β-CyD.

Drug	Additional Agent	Synthesis	Solubility *	Type of Cancer	In Vitro	Anticancer Effects	Reference
Curcumin	Chitosan	Stirring, lyophilization	279	Colon	HT-29	N.D.	[99]
Curcumin		Physical mixing/solvent evaporation	about 50–70	Breast	MCF-7	N.D.	[100]
Resveratrol		Coevaporation	438.6	Cervical	N.D.	N.D.	[104]

* The number represents the value when the solubility of the free drug is set to 1. N.D., not described.

**Table 4:** List of in vitro studies on anticancer drugs that demonstrated enhanced anti-cancer activity by forming complexes with HP-β-CyD.

Drug	Additional Agent	Synthesis	Solubility *	Type of Cancer	In Vitro	Anticancer Effects	Reference
Paclitaxel	Verapamil	Hot sonication, lyophilization	N.D.	Breast	MCF-7, MCF-7/ADR	Higher cytotoxicity and cellular uptake than PTX	[45]
Paclitaxel	Bcl-2 siRNA, L-arg	Facile synthesis, ligand exchange reaction	N.D.	Lung	A549	Increased cytotoxicity	[47]
Paclitaxel		Modified emulsion solvent evaporation	N.D.	Breast	MCF-7	Enhanced cytotoxicity	[57]
Paclitaxel	BM-HA-Man	Solution-stirring method	N.D.	Breast	MCF-7	Enhanced cytotoxicity	[58]
Paclitaxel	PLGA	Modified emulsion solvent evaporation	N.D.	Lung	A549	Improved anticancer activity and cellular uptake	[51]
Paclitaxel	PHB	Solvent evaporation, nanoprecipitation	N.D.	Breast, colon	MCF-7, MDA-MB-231, SW-620	Enhanced cytotoxicity, apoptosis, G2/M cell cycle arrest	[52]
Camptothecin		Sonication followed by magnetic stirring	N.D.	Breast, lung, liver, ovarian, neuroblastoma	AREc32, H-23, HepG2, A2780, SH-SY5Y	Enhanced cytotoxicity	[63]
9-nitro-camptothecin		Colyophilization	N.D.	Ovarian, breast, Cervical, mouse sarcoma	Skov-3, MCF-7, Hela, S180	Enhanced cytotoxicity	[66]
Doxorubicin + cisplatin		Sonication, lyophilization	N.D.	Osteosarcoma	KHOS/NP, MG-63	Enhanced cytotoxicity	[75]
Venetoclax	TPGS, EPI	Nanoprecipitation	N.D.	Breast (triple-negative)	4T1, MDA- MB-231	Enhanced cytotoxicity and cellular uptake	[90]
Resveratrol		N.D.	N.D.	Oral	HCPC1	Protection against the progression of carcinogenesis	[103]
C_60_		Coground	N.D.	Cervical	Hela	Growth inhibition	[113]
C_60_		Coground	N.D.	Cervical, lung	Hela, A549	Growth inhibition	[114]
Resiquimod (R848)	PLGA	Enhanced emulsification solvent-evaporation technique.	N.D.	Colon	MC38	Inhibition of tumor growth	[143]
Amygdalin		Ultrasonication, lyophilization	N.D.	Cervical	HeLa (CCL-2)	Growth inhibition	[150]
ADT-OH		Stirring	N.D.	Breast	MDA-MB-231, 4T1, MCF-7	Suppression of metastasis	[166]

* The number represents the value when the solubility of the free drug is set to 1. BM-HA-Man, hyaluronic acid grafted benzimidazole and mamnose; PLGA, poly(lactide-co-glycolide); PHB, poly-3-hydroxybutyrate; TPGS, D-α-tocopheryl polyethylene glycol succinate; EPI, epichlorohydrin; N.D., not described.

**Table 5:** List of in vivo studies using HP-β-CyD and anticancer drug complexes.

Drug	Additional Agent	Type of Cancer	In Vivo	Anticancer Effects	Other Effects	Reference
Paclitaxel		Breast	BALB/c nude (intratumoral)	Improved anticancer activity and cellular uptake	High bioavailability without worsening nephrotoxicity (i.v.)	[45]
Paclitaxel		Breast	U14 tumor (i.v.)	Enhanced cytotoxicity, tumor reduction effect		[57]
Paclitaxel	BM-HA-Man	Breast	4T1 tumor (i.v.)	Enhanced cytotoxicity, tumor reduction effect	Re-education of M2 to M1 macrophages	[58]
Paclitaxel		Lung	A549/T tumor (i.v.)	Enhanced cytotoxicity and cellular uptake, tumor reduction effect		[50]
Paclitaxel	PLGA	Lung	Kunming mice (i.v.)	Improved anticancer activity and cellular uptake	Superior biodistribution in lung	[51]
9-nitro-camptothecin		Ovarian, breast, cervical, sarcoma	S180 tumor (i.v. or i.m.)	Enhanced cytotoxicity, tumor reduction effect	Reduced toxicity compared to free 9-NC (cytopenia, liver and nephrotoxicity)	[66]
9-nitro-camptothecin	Transferrin, liposome	Ovarian, liver	S180 tumor (intratumoral)	Enhanced cytotoxicity, tumor reduction effect		[67]
Doxorubicin + cisplatin		Osteosarcoma	KHOS/NP tumor (injected around the cancer area)	Enhanced cytotoxicity, tumor reduction effect	Reduced cardiac and renal toxicities	[75]
Curcumin	Chitosan	Colon	Swiss albino (p.o.)	N.D.	Increased distribution to colon	[99]
Venetoclax	TPGS, EPI	Breast (triple- negative)	4T1 tumor (i.v.)	Enhanced cytotoxicity and cellular uptake, tumor reduction effect		[90]
Resveratrol		Oral	DMBA-induced oral cancer (p.o.)	Protection against the progression of carcinogenesis		[103]
Resveratrol		Cervical	Prevention & Treatment (Hela cells) (p.o.)	Inhibition of tumor growth	Decreased expression of HPV18-E6/E7, Increased expression of p53 and Rb1	[104]
C_60_		Cervical, lung cancer	mouse sarcoma S-80 (intratumoral)	Growth inhibition		[114]
Albendazole		Colorectal, prostate	HCT116 (i.p.)	Prolonged survival		[125]
Purinostat		B-cell lymphoma	SU-DHL-6 (i.v.)	Better anticancer effect and lower toxicity than Hyper-CVAD		[137]
Resiquimod (R848)	PLGA	Colon	MC38 (i.v.)	Inhibition of tumor growth and prolonged survival	Macrophage reprogramming	[143]
Thymoquinone		Lung	A549 (i.p.)	Enhanced cytotoxicity	NF-κB-mediated ferroptosis	[161]
ADT-OH		Breast	4T1 tumor (i.p.)	Suppression of metastasis		[166]

BM-HA-Man, hyaluronic acid grafted benzimidazole and mamnose; PLGA, poly(lactide-co-glycolide); TPGS, D-α-tocopheryl polyethylene glycol succinate; EPI, epichlorohydrin; p.o., per os; i.v., intravenous injection; i.p., intraperitoneal; N.D., not described.

**Table 6:** Examples of HP-β-CyD modified with folic acid.

	Guest	Additional Compound	In Vitro Model	In Vivo Model	Outcome	Reference
FA-HP-β-CyD	(-)	(-)	CML	CML	Apoptosis, autophagy, prolonged survival of CML mouse model	[203]
FA-HP-β-CyD	(-)	(-)	AML	AML	Apoptosis, autophagy, prolonged survival of AML mouse model	[204]
FA-HP-β-CyD	DXR	Bcl-2 siRNA	Breast cancer	N.D.	overcome MDR and enhance apoptosis	[207]

FA, folic acid; HP-β-CyD, hydroxypropyl-β-cyclodextrin; CML, chronic myeloid leukemia; AML, acute myeloid leukemia; DXR, doxorubicin; N.D., not done; MDR, multidrug resistance.

## Data Availability

No new data were created or analyzed in this study. Data sharing is not applicable to this article.

## References

[1] Davis M.E., Brewster M.E. (2004). Cyclodextrin-based pharmaceutics: Past, present and future. Nat. Rev. Drug Discov..

[2] Uekama K., Hirayama F., Irie T. (1998). Cyclodextrin Drug Carrier Systems. Chem. Rev..

[3] Szente L., Szejtli J. (1999). Highly soluble cyclodextrin derivatives: Chemistry, properties, and trends in development. Adv. Drug Deliv. Rev..

[4] Dahabra L., Broadberry G., Le Gresley A., Najlah M., Khoder M. (2021). Sunscreens Containing Cyclodextrin Inclusion Com plexes for Enhanced Efficiency: A Strategy for Skin Cancer Prevention. Molecules.

[5] Pitha J., Irie T., Sklar P.B., Nye J.S. (1988). Drug solubilizers to aid pharmacologists: Amorphous cyclodextrin derivatives. Life Sci..

[6] Loftsson T., Duchêne D. (2007). Cyclodextrins and their pharmaceutical applications. Int. J. Pharm..

[7] Kurkov S.V., Loftsson T. (2013). Cyclodextrins. Int. J. Pharm..

[8] Jansook P., Ogawa N., Loftsson T. (2018). Cyclodextrins: Structure, physicochemical properties and pharmaceutical applications. Int. J. Pharm..

[9] Muankaew C., Loftsson T. (2018). Cyclodextrin-Based Formulations: A Non-Invasive Platform for Targeted Drug Delivery. Basic. Clin. Pharmacol. Toxicol..

[10] Gould S., Scott R.C. (2005). 2-Hydroxypropyl-beta-cyclodextrin (HP-beta-CD): A toxicology review. Food Chem. Toxicol..

[11] Qu G., Han X., Ma L., Feng S., Li Y., Zhang X. (2025). Cyclodextrins as non-viral vectors in cancer theranostics: A review. Int. J. Biol. Macromol..

[12] Kfoury M., Landy D., Fourmentin S. (2018). Characterization of Cyclodextrin/Volatile Inclusion Complexes: A Review. Molecules.

[13] Cid-Samamed A., Rakmai J., Mejuto J.C., Simal-Gandara J., Astray G. (2022). Cyclodextrins inclusion complex: Preparation methods, analytical techniques and food industry applications. Food Chem..

[14] Szejtli J. (1998). Introduction and General Overview of Cyclodextrin Chemistry. Chem. Rev..

[15] Saokham P., Muankaew C., Jansook P., Loftsson T. (2018). Solubility of Cyclodextrins and Drug/Cyclodextrin Complexes. Molecules.

[16] Braga S.S. (2019). Cyclodextrins: Emerging Medicines of the New Millennium. Biomolecules.

[17] Liu B., Turley S.D., Burns D.K., Miller A.M., Repa J.J., Dietschy J.M. (2009). Reversal of defective lysosomal transport in NPC disease ameliorates liver dysfunction and neurodegeneration in the npc1-/- mouse. Proc. Natl. Acad. Sci. USA.

[18] Davidson C.D., Ali N.F., Micsenyi M.C., Stephney G., Renault S., Dobrenis K., Ory D.S., Vanier M.T., Walkley S.U. (2009). Chronic cyclodextrin treatment of murine Niemann-Pick C disease ameliorates neuronal cholesterol and glycosphingolipid storage and disease progression. PLoS ONE.

[19] Vite C.H., Bagel J.H., Swain G.P., Prociuk M., Sikora T.U., Stein V.M., O’Donnell P., Ruane T., Ward S., Crooks A. (2015). Intracisternal cyclodextrin prevents cerebellar dysfunction and Purkinje cell death in feline Niemann-Pick type C1 disease. Sci. Transl. Med..

[20] Ory D.S., Ottinger E.A., Farhat N.Y., King K.A., Jiang X., Weissfeld L., Berry-Kravis E., Davidson C.D., Bianconi S., Keener L.A. (2017). Intrathecal 2-hydroxypropyl-beta-cyclodextrin decreases neurological disease progression in Niemann-Pick disease, type C1: A non-randomised, open-label, phase 1-2 trial. Lancet.

[21] Hastings C., Liu B., Hurst B., Cox G.F., Hrynkow S. (2022). Intravenous 2-hydroxypropyl-β-cyclodextrin (Trappsol^®^ Cyclo™) demonstrates biological activity and impacts cholesterol metabolism in the central nervous system and peripheral tissues in adult subjects with Niemann-Pick Disease Type C1: Results of a phase 1 trial. Mol. Genet. Metab..

[22] Sharma R., Hastings C., Staretz-Chacham O., Raiman J., Paucar M., Spiegel R., Murray B., Hurst B., Liu B., Kjems L. (2023). Long-term administration of intravenous Trappsol^®^ Cyclo™ (HP-β-CD) results in clinical benefits and stabilization or slowing of disease progression in patients with Niemann-Pick disease type C1: Results of an international 48-week Phase I/II trial. Mol. Genet. Metab. Rep..

[23] Klein A.D., Eden E.R., Zanlungo S. (2025). Treating Niemann-Pick C lysosomal storage: Approved and emerging approaches. Trends Mol. Med..

[24] Lin Y.C., Hu S.C., Huang P.H., Lin T.C., Yen F.L. (2020). Electrospun Resveratrol-Loaded Polyvinylpyrrolidone/Cyclodextrin Nanofibers and Their Biomedical Applications. Pharmaceutics.

[25] Zhang M., Li J., Jia W., Chao J., Zhang L. (2009). Theoretical and experimental study of the inclusion complexes of ferulic acid with cyclodextrins. Supramol. Chem..

[26] Wang J., Cao Y., Sun B., Wang C. (2011). Characterisation of inclusion complex of trans-ferulic acid and hydroxypropyl-β-cyclodextrin. Food Chem..

[27] Hsu C.M., Yu S.C., Tsai F.J., Tsai Y. (2019). Characterization of in vitro and in vivo bioactivity of a ferulic acid-2-Hydroxypropyl-β-cyclodextrin inclusion complex. Colloids Surf. B Biointerfaces.

[28] Mercader-Ros M.T., Lucas-Abellán C., Fortea M.I., Gabaldón J.A., Núñez-Delicado E. (2010). Effect of HP-β-cyclodextrins complexation on the antioxidant activity of flavonols. Food Chem..

[29] de Oliveira M.R., Nabavi S.F., Habtemariam S., Erdogan Orhan I., Daglia M., Nabavi S.M. (2015). The effects of baicalein and baicalin on mitochondrial function and dynamics: A review. Pharmacol. Res..

[30] Kim H., Yiluo H., Park S., Lee J.Y., Cho E., Jung S. (2016). Characterization and Enhanced Antioxidant Activity of the Cysteinyl β-Cyclodextrin-Baicalein Inclusion Complex. Molecules.

[31] Yan T., Ji M., Sun Y., Yan T., Zhao J., Zhang H., Wang Z. (2019). Preparation and characterization of baicalein/hydroxypropyl-β- cyclodextrin inclusion complex for enhancement of solubility, antioxidant activity and antibacterial activity using supercritical antisolvent technology. J. Incl. Phenom. Macrocycl. Chem..

[32] Jullian C., Moyano L., Yañez C., Olea-Azar C. (2007). Complexation of quercetin with three kinds of cyclodextrins: An antioxidant study. Spectrochim. Acta A Mol. Biomol. Spectrosc..

[33] Haddad R., Alrabadi N., Altaani B., Li T. (2022). Paclitaxel Drug Delivery Systems: Focus on Nanocrystals’ Surface Modifications. Polymers.

[34] Lee S., Seo D., Kim H.W., Jung S. (2001). Investigation of inclusion complexation of paclitaxel by cyclohenicosakis-(1-->2)-(beta-D-glucopyranosyl), by cyclic-(1-->2)-beta-D-glucans (cyclosophoraoses), and by cyclomaltoheptaoses (beta-cyclodextrins). Carbohydr. Res..

[35] Agüeros M., Ruiz-Gatón L., Vauthier C., Bouchemal K., Espuelas S., Ponchel G., Irache J.M. (2009). Combined hydroxypropyl-beta-cyclodextrin and poly(anhydride) nanoparticles improve the oral permeability of paclitaxel. Eur. J. Pharm. Sci..

[36] Agüeros M., Zabaleta V., Espuelas S., Campanero M.A., Irache J.M. (2010). Increased oral bioavailability of paclitaxel by its encapsulation through complex formation with cyclodextrins in poly(anhydride) nanoparticles. J. Control. Release.

[37] Fenyvesi F., Fenyvesi E., Szente L., Goda K., Bacso Z., Bacskay I., Varadi J., Kiss T., Molnar E., Janaky T. (2008). P-glycoprotein inhibition by membrane cholesterol modulation. Eur. J. Pharm. Sci..

[38] Ishikawa M., Yoshii H., Furuta T. (2005). Interaction of modified cyclodextrins with cytochrome P-450. Biosci. Biotechnol. Biochem..

[39] Calleja P., Espuelas S., Vauthier C., Ponchel G., Irache J.M. (2015). Controlled Release, Intestinal Transport, and Oral Bioavailablity of Paclitaxel Can be Considerably Increased Using Suitably Tailored Pegylated Poly(Anhydride) Nanoparticles. J. Pharm. Sci..

[40] Baek J.S., So J.W., Shin S.C., Cho C.W. (2012). Solid lipid nanoparticles of paclitaxel strengthened by hydroxypropyl-β-cyclodextrin as an oral delivery system. Int. J. Mol. Med..

[41] Baek J.S., Cho C.W. (2013). 2-Hydroxypropyl-β-cyclodextrin-modified SLN of paclitaxel for overcoming p-glycoprotein function in multidrug-resistant breast cancer cells. J. Pharm. Pharmacol..

[42] Baek J.S., Kim J.H., Park J.S., Cho C.W. (2015). Modification of paclitaxel-loaded solid lipid nanoparticles with 2-hydroxypropyl-β-cyclodextrin enhances absorption and reduces nephrotoxicity associated with intravenous injection. Int. J. Nanomed..

[43] Baek J.S., Kim B.S., Puri A., Kumar K., Cho C.W. (2016). Stability of paclitaxel-loaded solid lipid nanoparticles in the presence of 2-hydoxypropyl-β-cyclodextrin. Arch Pharm Res.

[44] Huang M., Liu G. (1999). The study of innate drug resistance of human hepatocellular carcinoma Bel7402 cell line. Cancer Lett..

[45] Baek J.S., Cho C.W. (2015). Controlled release and reversal of multidrug resistance by co-encapsulation of paclitaxel and verapamil in solid lipid nanoparticles. Int. J. Pharm..

[46] Tan J., Meng N., Fan Y., Su Y., Zhang M., Xiao Y., Zhou N. (2016). Hydroxypropyl-β-cyclodextrin-graphene oxide conjugates: Carriers for anti-cancer drugs. Mater. Sci. Eng. C Mater. Biol. Appl..

[47] Li J., Wang Y., Xue S., Sun J., Zhang W., Hu P., Ji L., Mao Z. (2016). Effective combination treatment of lung cancer cells by single vehicular delivery of siRNA and different anticancer drugs. Int. J. Nanomed..

[48] Kneidl B., Peller M., Winter G., Lindner L.H., Hossann M. (2014). Thermosensitive liposomal drug delivery systems: State of the art review. Int. J. Nanomed..

[49] Xu Y., Qiu L. (2015). Nonspecifically enhanced therapeutic effects of vincristine on multidrug-resistant cancers when coencapsulated with quinine in liposomes. Int. J. Nanomed..

[50] Shen Q., Shen Y., Jin F., Du Y.Z., Ying X.Y. (2020). Paclitaxel/hydroxypropyl-β-cyclodextrin complex-loaded liposomes for overcoming multidrug resistance in cancer chemotherapy. J. Liposome Res..

[51] Zheng K., Huang Z., Huang J., Liu X., Ban J., Huang X., Luo H., Chen Z., Xie Q., Chen Y. (2021). Effect of a 2-HP-β-Cyclodextrin Formulation on the Biological Transport and Delivery of Chemotherapeutic PLGA Nanoparticles. Drug Des. Devel Ther..

[52] Aslam A., Masood F., Perveen K., Berger M.R., Pervaiz A., Zepp M., Klika K.D., Yasin T., Hameed A. (2024). Preparation, characterization and evaluation of HPβCD-PTX/PHB nanoparticles for pH-responsive, cytotoxic and apoptotic properties. Int. J. Biol. Macromol..

[53] Lu Y., Chen Y., Gemeinhart R.A., Wu W., Li T. (2015). Developing nanocrystals for cancer treatment. Nanomedicine.

[54] Jacob S., Nair A.B. (2018). Cyclodextrin complexes: Perspective from drug delivery and formulation. Drug Dev. Res..

[55] Liu J., Tu L., Cheng M., Feng J., Jin Y. (2020). Mechanisms for oral absorption enhancement of drugs by nanocrystals. J. Drug Deliv. Sci. Technol..

[56] Haddad R., Alrabadi N., Altaani B., Masadeh M., Li T. (2022). Hydroxypropyl Beta Cyclodextrin as a Potential Surface Modifier for Paclitaxel Nanocrystals. AAPS PharmSciTech.

[57] Yan C., Liang N., Li Q., Yan P., Sun S. (2019). Biotin and arginine modified hydroxypropyl-β-cyclodextrin nanoparticles as novel drug delivery systems for paclitaxel. Carbohydr. Polym..

[58] Fu J., Zhao W., Liang N., Sun S. (2025). Functionalized hydroxypropyl-β-cyclodextrin inclusion complex for combined tumor therapy through intelligent delivery of paclitaxel and polarization of M2-like tumor associated macrophages. Colloids Surf. B Biointerfaces.

[59] Hevener K., Verstak T.A., Lutat K.E., Riggsbee D.L., Mooney J.W. (2018). Recent developments in topoisomerase-targeted cancer chemotherapy. Acta Pharm. Sin. B.

[60] Venkatachalam A., Kaufmann S.H. (2025). Targeting DNA Topoisomerase I for the Treatment of Cancer: Past, Present and Future. J. Mol. Biol..

[61] Shen G., Li S., Zhu Y., Xu Z., Liu X., Lv C., Xing Z., Cui L., Li W. (2025). Recent progress in topoisomerase inhibitors as anticancer agents: Research and design strategies for Topo I and II inhibitors via structural optimization. Bioorg. Chem..

[62] Berrada M., Serreqi A., Dabbarh F., Owusu A., Gupta A., Lehnert S. (2005). A novel non-toxic camptothecin formulation for cancer chemotherapy. Biomaterials.

[63] González-Ruiz V., Cores Á., Martín-Cámara O., Orellana K., Cervera-Carrascón V., Michalska P., Olives A.I., León R., Martín M.A., Menéndez J.C. (2021). Enhanced Stability and Bioactivity of Natural Anticancer Topoisomerase I Inhibitors through Cyclodextrin Complexation. Pharmaceutics.

[64] Giovanella B.C., Stehlin J.S., Hinz H.R., Kozielski A.J., Harris N.J., Vardeman D.M. (2002). Preclinical evaluation of the anticancer activity and toxicity of 9-nitro-20(S)-camptothecin (Rubitecan). Int. J. Oncol..

[65] Jiang Y., Sha X., Zhang W., Fang X. (2010). Complex of 9-nitro-camptothecin in hydroxypropyl-beta-cyclodextrin: In vitro and in vivo evaluation. Int. J. Pharm..

[66] Jiang Y., Jiang X., Law K., Chen Y., Gu J., Zhang W., Xin H., Sha X., Fang X. (2011). Enhanced anti-tumor effect of 9-nitro-camptothecin complexed by hydroxypropyl-β-cyclodextrin and safety evaluation. Int. J. Pharm..

[67] Chen J., Lu S., Gu W., Peng P., Dong J., Xu F., Yang X., Xiong Z., Yang X. (2015). Characterization of 9-nitrocamptothecin-in-cyclodextrin-in-liposomes modified with transferrin for the treating of tumor. Int. J. Pharm..

[68] Moruno-Manchon J.F., Uzor N.E., Kesler S.R., Wefel J.S., Townley D.M., Nagaraja A.S., Pradeep S., Mangala L.S., Sood A.K., Tsvetkov A.S. (2016). TFEB ameliorates the impairment of the autophagy-lysosome pathway in neurons induced by doxorubicin. Aging.

[69] Coisne C., Tilloy S., Monflier E., Wils D., Fenart L., Gosselet F. (2016). Cyclodextrins as Emerging Therapeutic Tools in the Treatment of Cholesterol-Associated Vascular and Neurodegenerative Diseases. Molecules.

[70] Moruno-Manchon J.F., Uzor N.E., Kesler S.R., Wefel J.S., Townley D.M., Nagaraja A.S., Pradeep S., Mangala L.S., Sood A.K., Tsvetkov A.S. (2018). Peroxisomes contribute to oxidative stress in neurons during doxorubicin-based chemotherapy. Mol. Cell Neurosci..

[71] Durço A.O., Souza D.S., Rhana P., Costa A.D., Marques L.P., Santos L., de Souza Araujo A.A., de Aragão Batista M.V., Roman-Campos D., Santos M. (2023). d-Limonene complexed with cyclodextrin attenuates cardiac arrhythmias in an experimental model of doxorubicin-induced cardiotoxicity: Possible involvement of calcium/calmodulin-dependent protein kinase type II. Toxicol. Appl. Pharmacol..

[72] Durço A.O., Souza D.S., Lima Conceição M.R., Beserra S.D.S., Teixeira da Fonseca J.L., Loch L., Silva M.A., Pereira de Oliveira A., Araujo A.A.S., Lins de Vasconcelos C.M. (2025). D-limonene complexed with hydroxypropyl-β-cyclodextrin prevents impaired cardiac excitation-contraction coupling by reducing oxidative stress and cardiac apoptosis in an animal model of cardiotoxicity induced by doxorubicin. Chem. Biol. Interact..

[73] Yoo Y., Yoon S.J., Kim S.Y., Lee D.W., Um S., Hyun H., Hong S.O., Yang D.H. (2018). A local drug delivery system based on visible light-cured glycol chitosan and doxorubicin⋅hydrochloride for thyroid cancer treatment In Vitro and In Vivo. Drug Deliv..

[74] Hyun H., Park M.H., Jo G., Kim S.Y., Chun H.J., Yang D.H. (2019). Photo-Cured Glycol Chitosan Hydrogel for Ovarian Cancer Drug Delivery. Mar. Drugs.

[75] Yoon S.J., Moon Y.J., Chun H.J., Yang D.H. (2019). Doxorubicin·Hydrochloride/Cisplatin-Loaded Hydrogel/Nanosized (2-Hydroxypropyl)-Beta-Cyclodextrin Local Drug-Delivery System for Osteosarcoma Treatment In Vivo. Nanomaterials.

[76] Christou A., Parisis N.A., Tzakos A.G., Gerothanassis I.P., Goulas V. (2024). Optimization of β-cyclodextrin based ultrasound- assisted extraction: A green strategy to enhance the extraction of bioactive compounds from taro leaf byproduct. Sustain. Chem. Pharm..

[77] Niu W., Xu L., Li J., Zhai Y., Sun Z., Shi W., Jiang Y., Ma C., Lin H., Guo Y. (2020). Polyphyllin II inhibits human bladder cancer migration and invasion by regulating EMT-associated factors and MMPs. Oncol. Lett..

[78] Li J., Ma W., Cheng X., Zhang X., Xie Y., Ji Z., Wu S. (2020). Activation of FOXO3 pathway is involved in polyphyllin I-induced apoptosis and cell cycle arrest in human bladder cancer cells. Arch. Biochem. Biophys..

[79] Liu J., Liu Y.X., Song Y.S., Liu C.K., Yu F.X., Liu G., Xu T.R., Sang J. (2025). Targeting GRB2-Akt-SREBP1 signaling axis by saponins from Paris polyphylla Smith var. yunnanensis (Franch.) Hand.-Mazz to overcome cisplatin resistance in bladder cancer. J. Ethnopharmacol..

[80] Scully M.A., Wilkins D.E., Dang M.N., Hoover E.C., Aboeleneen S.B., Day E.S. (2023). Cancer Cell Membrane Wrapped Nanoparticles for the Delivery of a Bcl-2 Inhibitor to Triple-Negative Breast Cancer. Mol. Pharm..

[81] Inao T., Iida Y., Moritani T., Okimoto T., Tanino R., Kotani H., Harada M. (2018). Bcl-2 inhibition sensitizes triple-negative human breast cancer cells to doxorubicin. Oncotarget.

[82] Alhoshani A., Alatawi F.O., Al-Anazi F.E., Attafi I.M., Zeidan A., Agouni A., El Gamal H.M., Shamoon L.S., Khalaf S., Korashy H.M. (2020). BCL-2 Inhibitor Venetoclax Induces Autophagy-Associated Cell Death, Cell Cycle Arrest, and Apoptosis in Human Breast Cancer Cells. OncoTargets Ther..

[83] Rodenas M.C., Peñas-Martínez J., Pardo-Sánchez I., Zaragoza-Huesca D., Ortega-Sabater C., Peña-García J., Espín S., Ricote G., Montenegro S., Ayala-De La Peña F. (2023). Venetoclax is a potent hepsin inhibitor that reduces the metastatic and prothrombotic phenotypes of hepsin-expressing colorectal cancer cells. Front. Mol. Biosci..

[84] Bala Tannan N., Manzari M.T., Herviou L., Da Silva Ferreira M., Hagen C., Kiguchi H., Manova-Todorova K., Seshan V., de Stanchina E., Heller D.A. (2021). Tumor-targeted nanoparticles improve the therapeutic index of BCL2 and MCL1 dual inhibition. Blood.

[85] Liang B., Jiang D., Pan L., Xiong F., Feng S., Wu S., Ye H., Yu Z., Shi C., Gao S. (2022). Lipase-triggered drug release from BCL2 inhibitor ABT-199-loaded nanoparticles to elevate anti-leukemic activity through enhanced drug targeting on the mitochondrial membrane. Acta Biomater..

[86] Griffin J., Wu Y., Mu Q., Li X., Ho R.J.Y. (2023). Design and Characterization of a Novel Venetoclax-Zanubrutinib Nano-Combination for Enhancing Leukemic Cell Uptake and Long-Acting Plasma Exposure. Pharmaceutics.

[87] Adamo F.M., Silva Barcelos E.C., De Falco F., Dorillo E., Rompietti C., Sorcini D., Stella A., Del Papa B., Baldoni S., Esposito A. (2023). Therapeutic Targeting Potential of Novel Silver Nanoparticles Coated with Anti-CD20 Antibody against Chronic Lymphocytic Leukemia. Cancers.

[88] Yang J., Zhang P., Mao Y., Chen R., Cheng R., Li J., Sun H., Deng C., Zhong Z. (2024). CXCR4-Mediated Codelivery of FLT3 and BCL-2 Inhibitors for Enhanced Targeted Combination Therapy of FLT3-ITD Acute Myeloid Leukemia. Biomacromolecules.

[89] Rajana N., Chary P.S., Pooja Y.S., Bhavana V., Singh H., Guru S.K., Singh S.B., Mehra N.K. (2024). Quality by design approach-based fabrication and evaluation of self-nanoemulsifying drug delivery system for improved delivery of venetoclax. Drug Deliv. Transl. Res..

[90] Chandani S., Dighe S., Katari O., Yadav V., Jain S. (2025). Crosslinked hydroxypropyl-beta-cyclodextrin nanoparticles for improved efficacy of venetoclax against triple negative breast cancer. Int. J. Pharm..

[91] Patil S.K., Chary P.S., Maddipatla S., Madhavi Y.V., Singothu S., Bhandari V., Pardhi E., Bansal K.K., Mehra N.K. (2025). Development of venetoclax with 2-hydroxypropyl-beta-cyclodextrin inclusion complex for improved bioavailability. J. Biomol. Struct. Dyn..

[92] Fang J.Y., Hung C.F., Chiu H.C., Wang J.J., Chan T.F. (2003). Efficacy and irritancy of enhancers on the in-vitro and in-vivo percutaneous absorption of curcumin. J. Pharm. Pharmacol..

[93] Tomeh M.A., Hadianamrei R., Zhao X. (2019). A Review of Curcumin and Its Derivatives as Anticancer Agents. Int. J. Mol. Sci..

[94] Kunnumakkara A.B., Bordoloi D., Padmavathi G., Monisha J., Roy N.K., Prasad S., Aggarwal B.B. (2017). Curcumin, the golden nutraceutical: Multitargeting for multiple chronic diseases. Br. J. Pharmacol..

[95] Kunnumakkara A.B., Harsha C., Banik K., Vikkurthi R., Sailo B.L., Bordoloi D., Gupta S.C., Aggarwal B.B. (2019). Is curcumin bioavailability a problem in humans: Lessons from clinical trials. Expert. Opin. Drug Metab. Toxicol..

[96] Guo L.D., Chen X.J., Hu Y.H., Yu Z.J., Wang D., Liu J.Z. (2013). Curcumin inhibits proliferation and induces apoptosis of human colorectal cancer cells by activating the mitochondria apoptotic pathway. Phytother. Res..

[97] Lim T.G., Lee S.Y., Huang Z., Lim D.Y., Chen H., Jung S.K., Bode A.M., Lee K.W., Dong Z. (2014). Curcumin suppresses proliferation of colon cancer cells by targeting CDK2. Cancer Prev. Res..

[98] Johnson J.J., Mukhtar H. (2007). Curcumin for chemoprevention of colon cancer. Cancer Lett..

[99] Jyoti K., Bhatia R.K., Martis E.A.F., Coutinho E.C., Jain U.K., Chandra R., Madan J. (2016). Soluble curcumin amalgamated chitosan microspheres augmented drug delivery and cytotoxicity in colon cancer cells: In vitro and in vivo study. Colloids Surf. B Biointerfaces.

[100] Abdelkader H., Fatease A.A., Fathalla Z., Shoman M.E., Abou-Taleb H.A., Abourehab M.A.S. (2022). Design, Preparation and Evaluation of Supramolecular Complexes with Curcumin for Enhanced Cytotoxicity in Breast Cancer Cell Lines. Pharmaceutics.

[101] Jang M., Cai L., Udeani G.O., Slowing K.V., Thomas C.F., Beecher C.W., Fong H.H., Farnsworth N.R., Kinghorn A.D., Mehta R.G. (1997). Cancer chemopreventive activity of resveratrol, a natural product derived from grapes. Science.

[102] Kundu J.K., Surh Y.J. (2008). Cancer chemopreventive and therapeutic potential of resveratrol: Mechanistic perspectives. Cancer Lett..

[103] Berta G.N., Salamone P., Sprio A.E., Di Scipio F., Marinos L.M., Sapino S., Carlotti M.E., Cavalli R., Di Carlo F. (2010). Chemo prevention of 7,12-dimethylbenz[a]anthracene (DMBA)-induced oral carcinogenesis in hamster cheek pouch by topical application of resveratrol complexed with 2-hydroxypropyl-beta-cyclodextrin. Oral Oncol..

[104] Hao X., Sun X., Zhu H., Xie L., Wang X., Jiang N., Fu P., Sang M. (2021). Hydroxypropyl-β-Cyclodextrin-Complexed Resveratrol Enhanced Antitumor Activity in a Cervical Cancer Model: In Vivo Analysis. Front. Pharmacol..

[105] Lin H.S., Zhang W., Go M.L., Choo Q.Y., Ho P.C. (2010). Determination of Z-3,5,4′-trimethoxystilbene in rat plasma by a simple HPLC method: Application in a pre-clinical pharmacokinetic study. J. Pharm. Biomed. Anal..

[106] Lin H.S., Sviripa V.M., Watt D.S., Liu C., Xiang T.X., Anderson B.D., Ong P.S., Ho P.C. (2013). Quantification of trans-2,6-difluoro-4′-N,N-dimethylaminostilbene in rat plasma: Application to a pharmacokinetic study. J. Pharm. Biomed. Anal..

[107] Chen W., Yeo S.C., Elhennawy M.G., Xiang X., Lin H.S. (2015). Determination of naturally occurring resveratrol analog trans-4,4′-dihydroxystilbene in rat plasma by liquid chromatography-tandem mass spectrometry: Application to a pharmacokinetic study. Anal. Bioanal. Chem..

[108] Navarro-Orcajada S., Conesa I., Matencio A., García-Carmona F., López-Nicolás J.M. (2022). Molecular encapsulation and bioactivity of gnetol, a resveratrol analogue, for use in foods. J. Sci. Food Agric..

[109] Radeva L., Yordanov Y., Spassova I., Kovacheva D., Tzankova V., Yoncheva K. (2024). Double Encapsulation of Resveratrol and Doxorubicin in Composite Nanogel-An Opportunity to Reduce Cardio- and Neurotoxicity of Doxorubicin. Gels.

[110] Du Y., Zhao J., Li S., Yuan H. (2025). Application of photodynamic activation of prodrugs combined with phototherapy in tumor treatment. Mol. Cancer.

[111] Nagano T., Arakane K., Ryu A., Masunaga T., Shinmoto K., Mashiko S., Hirobe M. (1994). Comparison of singlet oxygen production efficiency of C60 with other photosensitizers, based on 1268 nm emission. Chem Pharm. Bull..

[112] Iohara D., Hirayama F., Higashi K., Yamamoto K., Uekama K. (2011). Formation of stable hydrophilic C60 nanoparticles by 2-hydroxypropyl-β-cyclodextrin. Mol. Pharm..

[113] Iohara D., Hiratsuka M., Hirayama F., Takeshita K., Motoyama K., Arima H., Uekama K. (2012). Evaluation of photodynamic activity of C60/2-hydroxypropyl-β-cyclodextrin nanoparticles. J. Pharm. Sci..

[114] Altaf A., Aldawsari H., Banjar Z.M., Iohara D., Anraku M., Uekama K., Hirashima F. (2014). Potential use of C60/2-hydroxypropyl-β-cyclodextrin nanoparticles as a new photosensitizer in the treatment of cancer. Int. J. Photoenergy.

[115] Yuan B., Yang R., Ma Y., Zhou S., Zhang X., Liu Y. (2017). A systematic review of the active saikosaponins and extracts isolated from Radix Bupleuri and their applications. Pharm. Biol..

[116] Hsu Y.L., Kuo P.L., Lin C.C. (2004). The proliferative inhibition and apoptotic mechanism of Saikosaponin D in human non-small cell lung cancer A549 cells. Life Sci..

[117] Lu C.N., Yuan Z.G., Zhang X.L., Yan R., Zhao Y.Q., Liao M., Chen J.X. (2012). Saikosaponin a and its epimer saikosaponin d exhibit anti-inflammatory activity by suppressing activation of NF-κB signaling pathway. Int. Immunopharmacol..

[118] Hu S.C., Lai Y.C., Lin C.L., Tzeng W.S., Yen F.L. (2019). Inclusion complex of saikosaponin-d with hydroxypropyl-β-cyclodextrin: Improved physicochemical properties and anti-skin cancer activity. Phytomedicine.

[119] Qiu N., Cheng X., Wang G., Wang W., Wen J., Zhang Y., Song H., Ma L., Wei Y., Peng A. (2014). Inclusion complex of barbigerone with hydroxypropyl-β-cyclodextrin: Preparation and in vitro evaluation. Carbohydr. Polym..

[120] Gao X., Cen L., Li F., Wen R., Yan H., Yao H., Zhu S. (2018). Oral administration of indole substituted dipyrido[2,3-d]pyrimidine derivative exhibits anti-tumor activity via inhibiting AKT and ERK1/2 on hepatocellular carcinoma. Biochem. Biophys. Res. Commun..

[121] Gao X., Wei M., Shan W., Liu Q., Gao J., Liu Y., Zhu S., Yao H. (2019). An oral 2-hydroxypropyl-β-cyclodextrin-loaded spirooxindole-pyrrolizidine derivative restores p53 activity via targeting MDM2 and JNK1/2 in hepatocellular carcinoma. Pharmacol. Res..

[122] Pourgholami M.H., Woon L., Almajd R., Akhter J., Bowery P., Morris D.L. (2001). In vitro and in vivo suppression of growth of hepatocellular carcinoma cells by albendazole. Cancer Lett..

[123] Pourgholami M.H., Akhter J., Wang L., Lu Y., Morris D.L. (2005). Antitumor activity of albendazole against the human colorectal cancer cell line HT-29: In vitro and in a xenograft model of peritoneal carcinomatosis. Cancer Chemother. Pharmacol..

[124] Pourgholami M.H., Yan Cai Z., Lu Y., Wang L., Morris D.L. (2006). Albendazole: A potent inhibitor of vascular endothelial growth factor and malignant ascites formation in OVCAR-3 tumor-bearing nude mice. Clin. Cancer Res..

[125] Ehteda A., Galettis P., Chu S.W., Pillai K., Morris D.L. (2012). Complexation of albendazole with hydroxypropyl-beta-cyclodextrin significantly improves its pharmacokinetic profile, cell cytotoxicity and antitumor efficacy in nude mice. Anticancer. Res..

[126] DeBord M.A., Southerland M.R., Wagers P.O., Tiemann K.M., Robishaw N.K., Whiddon K.T., Konopka M.C., Tessier C.A., Shriver L.P., Paruchuri S. (2017). Synthesis, characterization, In Vitro SAR and in vivo evaluation of N,N’bisnaphthylmethyl 2-alkyl substituted imidazolium salts against NSCLC. Bioorg. Med. Chem. Lett..

[127] Fenyvesi F., Nguyen T.L.P., Haimhoffer Á., Rusznyák Á., Vasvári G., Bácskay I., Vecsernyés M., Ignat S.R., Dinescu S., Costache M. (2020). Cyclodextrin Complexation Improves the Solubility and Caco-2 Permeability of Chrysin. Materials.

[128] Noh E.M., Park Y.J., Kim J.M., Kim M.S., Kim H.R., Song H.K., Hong O.Y., So H.S., Yang S.H., Kim J.S. (2015). Fisetin regulates TPA-induced breast cell invasion by suppressing matrix metalloproteinase-9 activation via the PKC/ROS/MAPK pathways. Eur. J. Pharmacol..

[129] Adhami V.M., Syed D.N., Khan N., Mukhtar H. (2012). Dietary flavonoid fisetin: A novel dual inhibitor of PI3K/Akt and mTOR for prostate cancer management. Biochem. Pharmacol..

[130] Maurya B.K., Trigun S.K. (2016). Fisetin Modulates Antioxidant Enzymes and Inflammatory Factors to Inhibit Aflatoxin-B1 Induced Hepatocellular Carcinoma in Rats. Oxid. Med. Cell. Longev..

[131] Suh Y., Afaq F., Johnson J.J., Mukhtar H. (2009). A plant flavonoid fisetin induces apoptosis in colon cancer cells by inhibition of COX2 and Wnt/EGFR/NF-kappaB-signaling pathways. Carcinogenesis.

[132] Kang K.A., Piao M.J., Hyun J.W. (2015). Fisetin induces apoptosis in human nonsmall lung cancer cells via a mitochondria-mediated pathway. Vitr. Cell Dev. Biol. Anim..

[133] Yi C., Zhang Y., Yu Z., Xiao Y., Wang J., Qiu H., Yu W., Tang R., Yuan Y., Guo W. (2014). Melatonin enhances the anti-tumor effect of fisetin by inhibiting COX-2/iNOS and NF-kappaB/p300 signaling pathways. PLoS ONE.

[134] Chou R.H., Hsieh S.C., Yu Y.L., Huang M.H., Huang Y.C., Hsieh Y.H. (2013). Fisetin inhibits migration and invasion of human cervical cancer cells by down-regulating urokinase plasminogen activator expression through suppressing the p38 MAPK- dependent NF-kappaB signaling pathway. PLoS ONE.

[135] Tripathi R., Samadder T., Gupta S., Surolia A., Shaha C. (2011). Anticancer activity of a combination of cisplatin and fisetin in embryonal carcinoma cells and xenograft tumors. Mol. Cancer Ther..

[136] Kadari A., Gudem S., Kulhari H., Bhandi M.M., Borkar R.M., Kolapalli V.R., Sistla R. (2017). Enhanced oral bioavailability and anticancer efficacy of fisetin by encapsulating as inclusion complex with HPβCD in polymeric nanoparticles. Drug Deliv..

[137] Zhu Z., Wen J., Xu Y., Pei H., Li D., Tang M., Bai P., He J., Yang Z., Chen L. (2022). Therapeutic efficacy of an injectable formulation of purinostat mesylate in SU-DHL-6 tumour model. Ann. Med..

[138] Yang L., Qiu Q., Tang M., Wang F., Yi Y., Yi D., Yang Z., Zhu Z., Zheng S., Yang J. (2019). Purinostat Mesylate Is a Uniquely Potent and Selective Inhibitor of HDACs for the Treatment of BCR-ABL-Induced B-Cell Acute Lymphoblastic Leukemia. Clin. Cancer Res..

[139] Jiang L., Liu X., Xuan G. (2020). Preparation of pH-Sensitive β-Cyclodextrin Derivatives and Evaluation of Their Drug-Loading Properties. IOP Conf. Ser. Mater. Sci. Eng..

[140] Dietsch G.N., Lu H., Yang Y., Morishima C., Chow L.Q., Disis M.L., Hershberg R.M. (2016). Coordinated Activation of Toll-Like Receptor8 (TLR8) and NLRP3 by the TLR8 Agonist, VTX-2337, Ignites Tumoricidal Natural Killer Cell Activity. PLoS ONE.

[141] Rook A.H., Gelfand J.M., Wysocka M., Troxel A.B., Benoit B., Surber C., Elenitsas R., Buchanan M.A., Leahy D.S., Watanabe R. (2015). Topical resiquimod can induce disease regression and enhance T-cell effector functions in cutaneous T-cell lymphoma. Blood.

[142] Varshney D., Qiu S.Y., Graf T.P., McHugh K.J. (2021). Employing Drug Delivery Strategies to Overcome Challenges Using TLR7/8 Agonists for Cancer Immunotherapy. AAPS J..

[143] Yuan H., Gui H., Chen S., Zhu L., Wang C., Jing Q., Lv H., Wan Q., Wang S., Zhou S. (2024). Regulating Tumor-Associated Macrophage Polarization by Cyclodextrin-Modified PLGA Nanoparticles Loaded with R848 for Treating Colon Cancer. Int. J. Nanomed..

[144] Makarević J., Tsaur I., Juengel E., Borgmann H., Nelson K., Thomas C., Bartsch G., Haferkamp A., Blaheta R.A. (2016). Amygdalin delays cell cycle progression and blocks growth of prostate cancer cells In Vitro. Life Sci..

[145] Chang H.K., Shin M.S., Yang H.Y., Lee J.W., Kim Y.S., Lee M.H., Kim J., Kim K.H., Kim C.J. (2006). Amygdalin induces apoptosis through regulation of Bax and Bcl-2 expressions in human DU145 and LNCaP prostate cancer cells. Biol. Pharm. Bull..

[146] Kwon H.Y., Hong S.P., Hahn D.H., Kim J.H. (2003). Apoptosis induction of Persicae Semen extract in human promyelocytic leukemia (HL-60) cells. Arch. Pharm. Res..

[147] Abboud M.M., Al Awaida W., Alkhateeb H.H., Abu-Ayyad A.N. (2019). Antitumor Action of Amygdalin on Human Breast Cancer Cells by Selective Sensitization to Oxidative Stress. Nutr. Cancer.

[148] Park H.J., Yoon S.H., Han L.S., Zheng L.T., Jung K.H., Uhm Y.K., Lee J.H., Jeong J.S., Joo W.S., Yim S.V. (2005). Amygdalin inhibits genes related to cell cycle in SNU-C4 human colon cancer cells. World J. Gastroenterol..

[149] Makarević J., Rutz J., Juengel E., Kaulfuss S., Tsaur I., Nelson K., Pfitzenmaier J., Haferkamp A., Blaheta R.A. (2014). Amygdalin influences bladder cancer cell adhesion and invasion In Vitro. PLoS ONE.

[150] Meenatchi V., Narayanan K.B., Sood A., Han S.S. (2024). Formation of amygdalin/β-cyclodextrin derivatives inclusion complexes for anticancer activity assessment in human cervical carcinoma HeLa cell line. Int. J. Pharm..

[151] Lombardo L.J., Lee F.Y., Chen P., Norris D., Barrish J.C., Behnia K., Castaneda S., Cornelius L.A., Das J., Doweyko A.M. (2004). Discovery of N-(2-chloro-6-methyl- phenyl)-2-(6-(4-(2-hydroxyethyl)- piperazin-1-yl)-2-methylpyrimidin-4- ylamino)thi azole-5-carboxamide (BMS-354825), a dual Src/Abl kinase inhibitor with potent antitumor activity in preclinical assays. J. Med. Chem..

[152] Itamura H., Kubota Y., Shindo T., Ando T., Kojima K., Kimura S. (2017). Elderly Patients with Chronic Myeloid Leukemia Benefit from a Dasatinib Dose as Low as 20 mg. Clin. Lymphoma Myeloma Leuk..

[153] Cutrignelli A., Sanarica F., Lopalco A., Lopedota A., Laquintana V., Franco M., Boccanegra B., Mantuano P., De Luca A., Denora N. (2019). Dasatinib/HP-β-CD Inclusion Complex Based Aqueous Formulation as a Promising Tool for the Treatment of Paediatric Neuromuscular Disorders. Int. J. Mol. Sci..

[154] Sokač K., Vrban L., Liović M., Škorić I., Vianello R., Bregović N., Žižek K. (2025). Controlled release of dasatinib from cyclodextrin-based inclusion complexes by mechanochemistry: A computational and experimental study. Int. J. Pharm..

[155] Zhang S.S., Nagasaka M. (2021). Spotlight on Sotorasib (AMG 510) for KRAS (G12C) Positive Non-Small Cell Lung Cancer. Lung Cancer.

[156] Nakajima E.C., Drezner N., Li X., Mishra-Kalyani P.S., Liu Y., Zhao H., Bi Y., Liu J., Rahman A., Wearne E. (2022). FDA Approval Summary: Sotorasib for KRAS G12C-Mutated Metastatic NSCLC. Clin. Cancer Res..

[157] Patel H.J., Patel K. (2025). Biosurfactant stabilized nanosuspension of KRAS inhibitor—Sotorasib (AMG-510): Systematic optimization using quality by design approach. Colloids Surf. B Biointerfaces.

[158] Zhao Z.X., Li S., Liu L.X. (2024). Thymoquinone affects hypoxia-inducible factor-1α expression in pancreatic cancer cells via HSP90 and PI3K/AKT/mTOR pathways. World J. Gastroenterol..

[159] Taiyab A., Choudhury A., Haidar S., Yousuf M., Rathi A., Koul P., Chakrabarty A., Islam A., Shamsi A., Hassan M.I. (2024). Exploring MTH1 inhibitory potential of Thymoquinone and Baicalin for therapeutic targeting of breast cancer. Biomed. Pharmacother..

[160] Shakeel I., Haider S., Khan S., Ahmed S., Hussain A., Alajmi M.F., Chakrabarty A., Afzal M., Imtaiyaz Hassan M. (2024). Thymoquinone, artemisinin, and thymol attenuate proliferation of lung cancer cells as Sphingosine kinase 1 inhibitors. Biomed. Pharmacother..

[161] Zheng W.W., Ding J.X., Liang Y.X., Ye L. (2025). Hydroxypropyl-β-cyclodextrin/thymoquinone inclusion complex inhibits non-small cell lung cancer progression through NF-κB-mediated ferroptosis. Oncol. Rep..

[162] Andre F., Zielinski C.C. (2012). Optimal strategies for the treatment of metastatic triple-negative breast cancer with currently approved agents. Ann. Oncol..

[163] Won K.A., Spruck C. (2020). Triple-negative breast cancer therapy: Current and future perspectives (Review). Int. J. Oncol..

[164] Cai F., Xu H., Cao N., Zhang X., Liu J., Lu Y., Chen J., Yang Y., Cheng J., Hua Z.C. (2020). ADT-OH, a hydrogen sulfide-releasing donor, induces apoptosis and inhibits the development of melanoma in vivo by upregulating FADD. Cell Death Dis..

[165] Jia J., Wang Z., Zhang M., Huang C., Song Y., Xu F., Zhang J., Li J., He M., Li Y. (2020). SQR mediates therapeutic effects of H(2)S by targeting mitochondrial electron transport to induce mitochondrial uncoupling. Sci. Adv..

[166] Yu S., Cao Z., Cai F., Yao Y., Chang X., Wang X., Zhuang H., Hua Z.C. (2024). ADT-OH exhibits anti-metastatic activity on triple-negative breast cancer by combinatorial targeting of autophagy and mitochondrial fission. Cell Death Dis..

[167] Bom A., Bradley M., Cameron K., Clark J.K., Van Egmond J., Feilden H., MacLean E.J., Muir A.W., Palin R., Rees D.C. (2002). A novel concept of reversing neuromuscular block: Chemical encapsulation of rocuronium bromide by a cyclodextrin-based synthetic host. Angew. Chem. Int. Ed. Engl..

[168] Adam J.M., Bennett D.J., Bom A., Clark J.K., Feilden H., Hutchinson E.J., Palin R., Prosser A., Rees D.C., Rosair G.M. (2002). Cyclodextrin-derived host molecules as reversal agents for the neuromuscular blocker rocuronium bromide: Synthesis and structure-activity relationships. J. Med. Chem..

[169] Epemolu O., Bom A., Hope F., Mason R. (2003). Reversal of neuromuscular blockade and simultaneous increase in plasma rocuronium concentration after the intravenous infusion of the novel reversal agent Org 25969. Anesthesiology.

[170] Gijsenbergh F., Ramael S., Houwing N., van Iersel T. (2005). First human exposure of Org 25969, a novel agent to reverse the action of rocuronium bromide. Anesthesiology.

[171] Vanier M.T. (2010). Niemann-Pick disease type C. Orphanet J. Rare Dis..

[172] Griffin L.D., Gong W., Verot L., Mellon S.H. (2004). Niemann-Pick type C disease involves disrupted neurosteroidogenesis and responds to allopregnanolone. Nat. Med..

[173] Matsuo M., Togawa M., Hirabaru K., Mochinaga S., Narita A., Adachi M., Egashira M., Irie T., Ohno K. (2013). Effects of cyclodextrin in two patients with Niemann-Pick Type C disease. Mol. Genet. Metab..

[174] Matsuo M., Shraishi K., Wada K., Ishitsuka Y., Doi H., Maeda M., Mizoguchi T., Eto J., Mochinaga S., Arima H. (2014). Effects of intracerebroventricular administration of 2-hydroxypropyl-β-cyclodextrin in a patient with Niemann-Pick Type C disease. Mol. Genet. Metab. Rep..

[175] Megías-Vericat J.E., García-Robles A., Company-Albir M.J., Fernández-Megía M.J., Pérez-Miralles F.C., López-Briz E., Casanova B., Poveda J.L. (2017). Early experience with compassionate use of 2 hydroxypropyl-beta-cyclodextrin for Niemann-Pick type C disease: Review of initial published cases. Neurol. Sci..

[176] Sitarska D., Tylki-Szymańska A., Ługowska A. (2021). Treatment trials in Niemann-Pick type C disease. Metab. Brain Dis..

[177] Li H.Y., Appelbaum F.R., Willman C.L., Zager R.A., Banker D.E. (2003). Cholesterol-modulating agents kill acute myeloid leukemia cells and sensitize them to therapeutics by blocking adaptive cholesterol responses. Blood.

[178] Ghalaut V.S., Pahwa M.B., Sunita, Ghalaut P.S. (2006). Alteration in lipid profile in patients of chronic myeloid leukemia before and after chemotherapy. Clin. Chim. Acta.

[179] Mulas M.F., Abete C., Pulisci D., Pani A., Massidda B., Dessi S., Mandas A. (2011). Cholesterol esters as growth regulators of lymphocytic leukaemia cells. Cell Prolif..

[180] Rudling M., Gafvels M., Parini P., Gahrton G., Angelin B. (1998). Lipoprotein receptors in acute myelogenous leukemia: Failure to detect increased low-density lipoprotein (LDL) receptor numbers in cell membranes despite increased cellular LDL degradation. Am. J. Pathol..

[181] Vitols S., Norgren S., Juliusson G., Tatidis L., Luthman H. (1994). Multilevel regulation of low-density lipoprotein receptor and 3-hydroxy-3-methylglutaryl coenzyme A reductase gene expression in normal and leukemic cells. Blood.

[182] Brindisi M., Fiorillo M., Frattaruolo L., Sotgia F., Lisanti M.P., Cappello A.R. (2020). Cholesterol and Mevalonate: Two Metabolites Involved in Breast Cancer Progression and Drug Resistance through the ERRα Pathway. Cells.

[183] Ehmsen S., Pedersen M.H., Wang G., Terp M.G., Arslanagic A., Hood B.L., Conrads T.P., Leth-Larsen R., Ditzel H.J. (2019). Increased Cholesterol Biosynthesis Is a Key Characteristic of Breast Cancer Stem Cells Influencing Patient Outcome. Cell Rep..

[184] Dattilo R., Mottini C., Camera E., Lamolinara A., Auslander N., Doglioni G., Muscolini M., Tang W., Planque M., Ercolani C. (2020). Pyrvinium Pamoate Induces Death of Triple-Negative Breast Cancer Stem-Like Cells and Reduces Metastases through Effects on Lipid Anabolism. Cancer Res..

[185] Beckwitt C.H., Shiraha K., Wells A. (2018). Lipophilic statins limit cancer cell growth and survival, via involvement of Akt signaling. PLoS ONE.

[186] Beckwitt C.H., Clark A.M., Ma B., Whaley D., Oltvai Z.N., Wells A. (2018). Statins attenuate outgrowth of breast cancer metastases. Br. J. Cancer.

[187] Mollinedo F., Gajate C. (2015). Lipid rafts as major platforms for signaling regulation in cancer. Adv. Biol. Regul..

[188] Li B., Qin Y., Yu X., Xu X., Yu W. (2022). Lipid raft involvement in signal transduction in cancer cell survival, cell death and metastasis. Cell Prolif..

[189] Yokoo M., Kubota Y., Motoyama K., Higashi T., Taniyoshi M., Tokumaru H., Nishiyama R., Tabe Y., Mochinaga S., Sato A. (2015). 2-Hydroxypropyl-beta-Cyclodextrin Acts as a Novel Anticancer Agent. PLoS ONE.

[190] Wu Y., Zhao Y., He X., He Z., Wang T., Wan L., Chen L., Yan N. (2020). Hydroxypropyl-β-cyclodextrin attenuates the epithetlial-to-mesenchymal transition via endoplasmic reticulum stress in MDA-MB-231 breast cancer cells. Mol. Med. Rep..

[191] Zhao Y., He L., Wang T., Zhu L., Yan N. (2021). 2-Hydroxypropyl-β-cyclodextrin Regulates the Epithelial to Mesenchymal Transition in Breast Cancer Cells by Modulating Cholesterol Homeostasis and Endoplasmic Reticulum Stress. Metabolites.

[192] Saha S.T., Abdulla N., Zininga T., Shonhai A., Wadee R., Kaur M. (2023). 2-Hydroxypropyl-beta-cyclodextrin (HPbetaCD) as a Potential Therapeutic Agent for Breast Cancer. Cancers.

[193] Zhu M., Zhao Q., Zhang W., Xu H., Zhang B., Zhang S., Duan Y., Liao C., Yang X., Chen Y. (2023). Hydroxypropyl-beta-cyclodextrin inhibits the development of triple negative breast cancer by enhancing antitumor immunity. Int. Immunopharmacol..

[194] Bajracharya R., Song J.G., Patil B.R., Lee S.H., Noh H.M., Kim D.H., Kim G.L., Seo S.H., Park J.W., Jeong S.H. (2022). Functional ligands for improving anticancer drug therapy: Current status and applications to drug delivery systems. Drug Deliv..

[195] Anarjan F.S. (2019). Active targeting drug delivery nanocarriers: Ligands. Nano- Struct. Nano-Objects.

[196] Parker N., Turk M.J., Westrick E., Lewis J.D., Low P.S., Leamon C.P. (2005). Folate receptor expression in carcinomas and normal tissues determined by a quantitative radioligand binding assay. Anal. Biochem..

[197] Assaraf Y.G., Leamon C.P., Reddy J.A. (2014). The folate receptor as a rational therapeutic target for personalized cancer treatment. Drug Resist. Updat..

[198] Gonen N., Assaraf Y.G. (2012). Antifolates in cancer therapy: Structure, activity and mechanisms of drug resistance. Drug Resist. Updat..

[199] Onodera R., Motoyama K., Okamatsu A., Higashi T., Arima H. (2013). Potential use of folate-appended methyl-beta-cyclodextrin as an anticancer agent. Sci. Rep..

[200] Onodera R., Motoyama K., Tanaka N., Ohyama A., Okamatsu A., Higashi T., Kariya R., Okada S., Arima H. (2014). Involvement of autophagy in antitumor activity of folate-appended methyl-beta-cyclodextrin. Sci. Rep..

[201] Motoyama K., Onodera R., Tanaka N., Kameyama K., Higashi T., Kariya R., Okada S., Arima H. (2015). Evaluation of antitumor effects of folate-conjugated methyl-beta-cyclodextrin in melanoma. Biol. Pharm. Bull..

[202] Kameyama K., Motoyama K., Tanaka N., Yamashita Y., Higashi T., Arima H. (2017). Induction of mitophagy-mediated antitumor activity with folate-appended methyl-β-cyclodextrin. Int. J. Nanomed..

[203] Hoshiko T., Kubota Y., Onodera R., Higashi T., Yokoo M., Motoyama K., Kimura S. (2021). Folic Acid-Appended Hydroxypropyl-beta-Cyclodextrin Exhibits Potent Antitumor Activity in Chronic Myeloid Leukemia Cells via Autophagic Cell Death. Cancers.

[204] Kubota Y., Hoshiko T., Higashi T., Motoyama K., Okada S., Kimura S. (2023). Folate-Appended Hydroxypropyl-beta-Cyclodextrin Induces Autophagic Cell Death in Acute Myeloid Leukemia Cells. Int. J. Mol. Sci..

[205] Kubota Y., Kimura S. (2024). Current Understanding of the Role of Autophagy in the Treatment of Myeloid Leukemia. Int. J. Mol. Sci..

[206] Saito S., Koya Y., Kajiyama H., Yamashita M., Kikkawa F., Nawa A. (2020). Folate-appended cyclodextrin carrier targets ovarian cancer cells expressing the proton-coupled folate transporter. Cancer Sci..

[207] Li J.M., Zhang W., Su H., Wang Y.Y., Tan C.P., Ji L.N., Mao Z.W. (2015). Reversal of multidrug resistance in MCF-7/Adr cells by codelivery of doxorubicin and BCL2 siRNA using a folic acid-conjugated polyethylenimine hydroxypropyl-β-cyclodextrin nanocarrier. Int. J. Nanomed..

[208] Tanaka Y., Yamada Y., Ishitsuka Y., Matsuo M., Shiraishi K., Wada K., Uchio Y., Kondo Y., Takeo T., Nakagata N. (2015). Efficacy of 2-Hydroxypropyl-β-cyclodextrin in Niemann-Pick Disease Type C Model Mice and Its Pharmacokinetic Analysis in a Patient with the Disease. Biol. Pharm. Bull..

[209] Irie T., Uekama K. (1997). Pharmaceutical applications of cyclodextrins. III. Toxicological issues and safety evaluation. J. Pharm. Sci..

[210] Loftsson T. (2021). Cyclodextrins in Parenteral Formulations. J. Pharm. Sci..

[211] Mantik P., Xie M., Wong H., La H., Steigerwalt R.W., Devanaboyina U., Ganem G., Shih D., Flygare J.A., Fairbrother W.J. (2019). Cyclodextrin Reduces Intravenous Toxicity of a Model Compound. J. Pharm. Sci..

[212] Pitha J., Szente L. (1983). Rescue from hypervitaminosis A or potentiation of retinoid toxicity by different modes of cyclodextrin administration. Life Sci..

[213] Chien Y.H., Shieh Y.D., Yang C.Y., Lee N.C., Hwu W.L. (2013). Lung toxicity of hydroxypropyl-beta-cyclodextrin infusion. Mol. Genet. Metab..

[214] Ramirez C.M., Liu B., Taylor A.M., Repa J.J., Burns D.K., Weinberg A.G., Turley S.D., Dietschy J.M. (2010). Weekly cyclodextrin administration normalizes cholesterol metabolism in nearly every organ of the Niemann-Pick type C1 mouse and markedly prolongs life. Pediatr. Res..

[215] Muralidhar A., Borbon I.A., Esharif D.M., Ke W., Manacheril R., Daines M., Erickson R.P. (2011). Pulmonary function and pathology in hydroxypropyl-beta-cyclodextin-treated and untreated Npc1(-)/(-) mice. Mol. Genet. Metab..

[216] Ward S., O’Donnell P., Fernandez S., Vite C.H. (2010). 2-hydroxypropyl-beta-cyclodextrin raises hearing threshold in normal cats and in cats with Niemann-Pick type C disease. Pediatr. Res..

[217] Crumling M.A., Liu L., Thomas P.V., Benson J., Kanicki A., Kabara L., Halsey K., Dolan D., Duncan R.K. (2012). Hearing loss and hair cell death in mice given the cholesterol-chelating agent hydroxypropyl-beta-cyclodextrin. PLoS ONE.

[218] Cronin S., Lin A., Thompson K., Hoenerhoff M., Duncan R.K. (2015). Hearing Loss and Otopathology Following Systemic and Intracerebroventricular Delivery of 2-Hydroxypropyl-Beta-Cyclodextrin. J. Assoc. Res. Otolaryngol..

[219] Crumling M.A., King K.A., Duncan R.K. (2017). Cyclodextrins and Iatrogenic Hearing Loss: New Drugs with Significant Risk. Front. Cell Neurosci..

[220] Stella V.J., He Q. (2008). Cyclodextrins. Toxicol. Pathol..

[221] De Schaepdrijver L., Mariën D., Rhimi C., Voets M., van Heerden M., Lammens L. (2015). Juvenile animal testing of hydroxypro pyl-β-cyclodextrin in support of pediatric drug development. Reprod. Toxicol..

[222] Centini M., Maggiore M., Casolaro M., Andreassi M., Maffei Facino R., Anselmi C. (2007). Cyclodextrins as cosmetic delivery systems. J. Incl. Phenom. Macrocycl. Chem..

[223] Dickenmann M., Oettl T., Mihatsch M.J. (2008). Osmotic nephrosis: Acute kidney injury with accumulation of proximal tubular lysosomes due to administration of exogenous solutes. Am. J. Kidney Dis..

[224] Lin E.Y., Chen Y.S., Li Y.S., Chen S.R., Lee C.H., Huang M.H., Chuang H.M., Harn H.J., Yang H.H., Lin S.Z. (2020). Liposome Consolidated with Cyclodextrin Provides Prolonged Drug Retention Resulting in Increased Drug Bioavailability in Brain. Int. J. Mol. Sci..

[225] Hastings C., Vieira C., Liu B., Bascon C., Gao C., Wang R.Y., Casey A., Hrynkow S. (2019). Expanded access with intravenous hydroxypropyl-beta-cyclodextrin to treat children and young adults with Niemann-Pick disease type C1: A case report analysis. Orphanet J. Rare Dis..

[226] Soga M., Ishitsuka Y., Hamasaki M., Yoneda K., Furuya H., Matsuo M., Ihn H., Fusaki N., Nakamura K., Nakagata N. (2015). HPGCD outperforms HPBCD as a potential treatment for Niemann-Pick disease type C during disease modeling with iPS cells. Stem Cells.

[227] Yamada Y., Ishitsuka Y., Kondo Y., Nakahara S., Nishiyama A., Takeo T., Nakagata N., Motoyama K., Higashi T., Arima H. (2021). Differential mode of cholesterol inclusion with 2-hydroxypropyl-cyclodextrins increases safety margin in treatment of Niemann-Pick disease type C. Br. J. Pharmacol..

[228] Abdelkader H., Fathalla Z., Moharram H., Ali T.F.S., Pierscionek B. (2018). Cyclodextrin Enhances Corneal Tolerability and Reduces Ocular Toxicity Caused by Diclofenac. Oxid. Med. Cell. Longev..

[229] Rassu G., Fancello S., Roldo M., Malanga M., Szente L., Migheli R., Gavini E., Giunchedi P. (2020). Investigation of Cytotoxicity and Cell Uptake of Cationic Beta-Cyclodextrins as Valid Tools in Nasal Delivery. Pharmaceutics.

[230] Santos A.C., Costa D., Ferreira L., Guerra C., Pereira-Silva M., Pereira I., Peixoto D., Ferreira N.R., Veiga F. (2021). Cyclodextrin-based delivery systems for in vivo-tested anticancer therapies. Drug Deliv. Transl. Res..

